# Plasma Membrane-Targeted PIN Proteins Drive Shoot Development in a Moss

**DOI:** 10.1016/j.cub.2014.09.054

**Published:** 2014-12-01

**Authors:** Tom A. Bennett, Maureen M. Liu, Tsuyoshi Aoyama, Nicole M. Bierfreund, Marion Braun, Yoan Coudert, Ross J. Dennis, Devin O’Connor, Xiao Y. Wang, Chris D. White, Eva L. Decker, Ralf Reski, C. Jill Harrison

**Affiliations:** 1 Plant Sciences Department, University of Cambridge, Downing Street, Cambridge CB2 3EA, UK; 2 Sainsbury Laboratory, University of Cambridge, Bateman Street, Cambridge CB2 1LR, UK; 3 Faculty of Biology, University of Freiburg, Schänzlestraße 1, 79104 Freiburg, Germany; 4 BIOSS Centre for Biological Signalling Studies, 79104 Freiburg, Germany; 5 Freiburg Institute for Advanced Studies (FRIAS), 79104 Freiburg, Germany

## Abstract

**Background:**

Plant body plans arise by the activity of meristematic growing tips during development and radiated independently in the gametophyte (n) and sporophyte (2n) stages of the life cycle during evolution. Although auxin and its intercellular transport by PIN family efflux carriers are primary regulators of sporophytic shoot development in flowering plants, the extent of conservation in PIN function within the land plants and the mechanisms regulating bryophyte gametophytic shoot development are largely unknown.

**Results:**

We have found that treating gametophytic shoots of the moss *Physcomitrella patens* with exogenous auxins and auxin transport inhibitors disrupts apical function and leaf development. Two plasma membrane-targeted PIN proteins are expressed in leafy shoots, and *pin* mutants resemble plants treated with auxins or auxin transport inhibitors. PIN-mediated auxin transport regulates apical cell function, leaf initiation, leaf shape, and shoot tropisms in moss gametophytes. *pin* mutant sporophytes are sometimes branched, reproducing a phenotype only previously seen in the fossil record and in rare natural moss variants.

**Conclusions:**

Our results show that PIN-mediated auxin transport is an ancient, conserved regulator of shoot development.

## Highlights

• PIN proteins have polar plasma membrane localizations in the moss *Physcomitrella*
• PIN-mediated auxin transport drives gametophytic shoot development in *Physcomitrella*
• PIN-mediated auxin transport suppresses branching in *Physcomitrella* sporophytes

## Teaser

Shooting systems have undergone 450 million years of independent evolution in flowering plant sporophytes and bryophyte gametophytes. Bennett et al. show that PIN-mediated auxin transport regulates shoot development in both life cycle stages in a moss and identify potential roles for PIN proteins in the evolution of plant body plans.

## Introduction

Land plants evolved from freshwater algae with a haploid-dominant life cycle in which meiosis occurred straight after fertilization, and the colonization of land around 450 million years ago was accompanied by the innovation of a multicellular diploid body [1, 2, 3, 4]. Complex morphologies diversified independently in both the haploid (gametophyte) and diploid (sporophyte) life cycle stages in different plant groups during evolution [4, 5]. Bryophytes comprise a basal, gametophyte-dominant grade [6, 7, 8] with widely divergent thalloid, filamentous or shoot-like gametophytic forms, and the sporophyte comprises a single stem capped in a sporangium [2, 9, 10].The emergence of the vascular plant clade was associated with a shift to sporophyte dominance, a suite of sporophytic innovations including branching, and a gradual reduction in gametophyte size [4, 11, 12, 13]. The mechanisms underpinning architectural diversification in each life cycle stage are unknown, but the shared genetic toolkit available to land plants implicates conserved developmental mechanisms [14, 15].

One major candidate for such a conserved mechanism is the regulated intercellular transport of the plant hormone, auxin [16]. Most of our understanding of the key contribution of auxin transport to meristem function and shoot architecture comes from studies in flowering plants [17]. Pharmacological treatments that disrupt auxin transport across the multicellular apical dome inhibit leaf initiation [18], and in *Arabidopsis*, mutations in the auxin efflux carrier *PIN-FORMED1* (*PIN1*) gene cause similar defects [19]. Local application of auxin to naked apices is sufficient to induce leaf initiation, and such auxin maximum formation usually occurs as a result of the dynamic polar transport of auxin by PIN1 to foci on the meristem [18, 20, 21]. Distinct patterns of leaf initiation arise as a consequence of the self-organizing properties of the auxin transport system [22, 23]. Patterns of leaflet initiation [24], vein insertion in leaves [25], marginal ornamentation [26], and leaf growth [27] are similarly regulated by PIN-dependent auxin transport. Thus, PIN-mediated auxin transport acts as a major contributor to architectural diversity in flowering plants by modulating meristem function and leaf development.

Auxin transport assays and auxin transport inhibitor applications in the lycophyte *Selaginella kraussiana* have shown that auxin transport has conserved roles in sporophytic meristem function within the vascular plants [28, 29, 30, 31]. Several recent papers have considered the contributions of auxin and its transport to bryophyte development, using mosses as model systems [32, 33, 34, 35]. Bulk basipetal polar auxin transport has been demonstrated in moss sporophytes, and application of polar auxin transport inhibitors (PATIs) causes severe disruptions in development, resulting in the formation of multiple sporangia [32, 33]. These data suggest that polar auxin transport is a conserved regulator of sporophyte development, but the extent of conservation between the sporophyte and gametophyte generation is unclear. Although gametophytic auxin transport has been reported in ferns [36], mosses [37, 38], liverworts [39, 40], and charophyte algae [41], it has proved undetectable in the gametophytic shoots of mosses [32, 33]. As sporophytic and gametophytic shoots (gametophores) evolved independently, the convergent shoot morphologies of each generation could have arisen through the recruitment of distinct genetic pathways to regulate development in plant evolution [32, 33].

One hypothesis to account for the divergent auxin transport properties of sporophytic and gametophytic shooting systems in mosses is a divergence in PIN function between mosses and vascular plants or between generations in mosses. In *Arabidopsis*, PIN function depends on subcellular protein localizations; whereas PIN1–PIN4 and PIN7 (canonical PINs) are plasma membrane targeted and function in many developmental processes by regulating intercellular auxin transport, PIN5, PIN6, and PIN8 (noncanonical PINs) are ER targeted and are thought to regulate auxin homeostasis within cells [42, 43, 44]. The apparent functional divergence between canonical and noncanonical PINs reflects differences in protein structure between the two classes, and canonical PINs have a predicted intracellular domain with characteristic motifs involved in membrane targeting, which is greatly reduced in noncanonical PINs [45, 46]. The genome of the model moss *Physcomitrella patens* encodes four PIN proteins (PINA–PIND), whose localization has been assayed by heterologous expression assays in tobacco protoplasts. These suggested that PINA localizes to the ER and that PIND localizes in the cytosol, implying roles in intracellular auxin homeostasis rather than intercellular transport [34]. Although these data support the hypothesis that the absence of bulk basipetal auxin transport in moss gametophores could reflect a divergence in PIN function between mosses and flowering plants, they cannot account for the divergent auxin transport properties of moss sporophytes and gametophores. Furthermore, we have recently shown that vascular plant PIN proteins diversified from a single canonical ancestor and that three *Physcomitrella* PINs (PINA–PINC) have canonical structure, placing canonical PINs one likely ancestral type within the land plants [45]. The data above raise questions about the evolution of land plant PIN functions and the roles of auxin transport and PIN proteins in moss gametophore development.

Here, we show that *Physcomitrella* PINs are plasma membrane targeted and that PIN-mediated auxin transport regulates many aspects of gametophore development. *pin* mutants have greatly impaired fertility and striking sporophytic defects that are similar to published defects arising from treatment with auxin transport inhibitors. Our results show that PIN proteins are conserved auxin transport facilitators.

## Results

### Exogenously Applied Auxins Affect Meristem Function and Leaf Development

**Figure 1:**
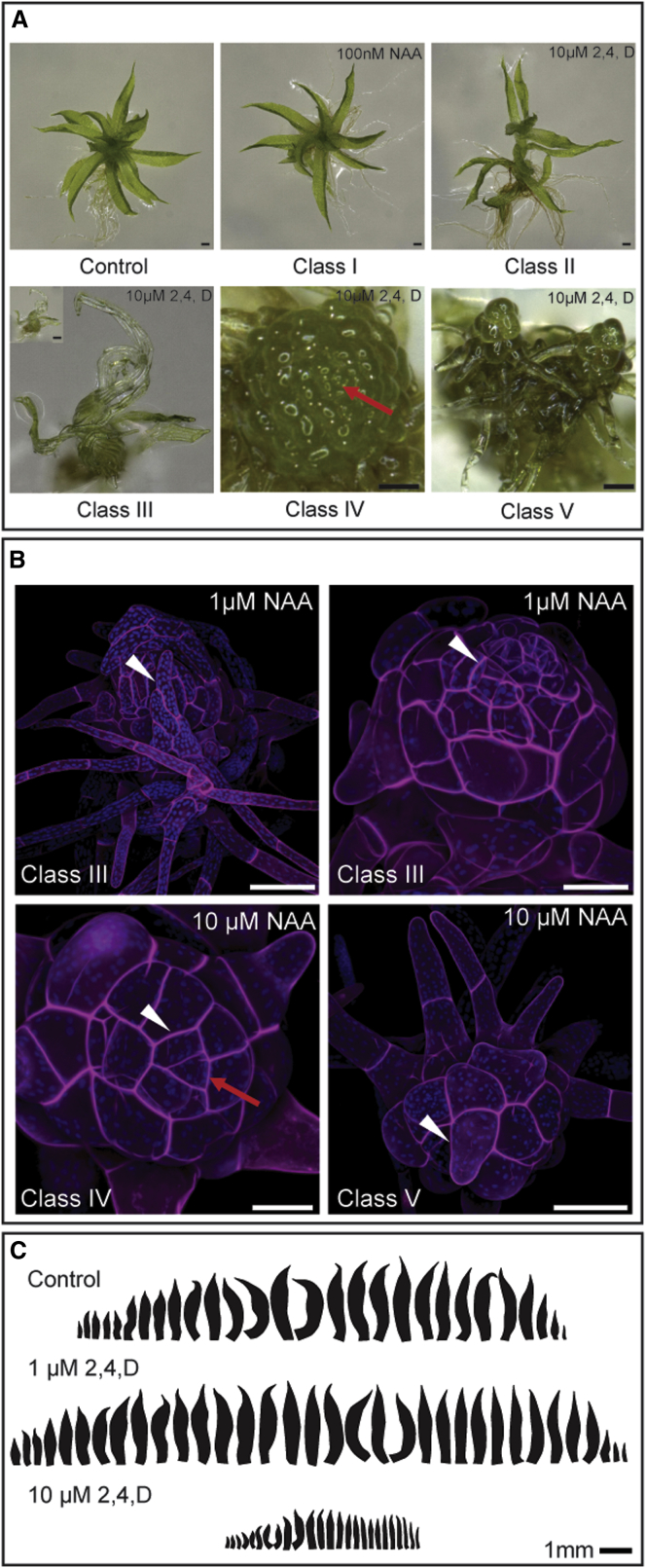
Treatment with Auxins Perturbs Leaf Development and Can Cause Meristem Arrest Plants were grown on BCD + ammonium tartrate (AT) medium for 3 weeks in continuous light at 23°C. (A) Developmental defects arising as a result of auxin treatments. Scale bars in untreated control, class I and class II, and inset for class III represent 200 μm; scale bars in class IV and class V represent 100 μm. Red arrow indicates the apical cell. (B) Confocal micrographs of class III–V buds showing severely stunted leaves (arrowheads in 1μM NAA treatments), a leaf progenitor cell and apical cell (arrowhead and arrow in class IV shoot), and a rhizoid terminating the shoot (arrowhead in class V shoot). Scale bars represent 50 μm. (C) Leaf series of plants grown on different auxin treatments. 1 μM 2,4-D mildly promotes leaf initiation and development, whereas 10 μM 2,4-D inhibits leaf initiation and development relative to controls.

To clarify the roles of auxin in moss gametophore development, we grew colonies on medium supplemented with auxins that have different biochemical properties: indoleacetic acid (IAA), naphthylacetic acid (NAA), and 2,4-dichlorophenoxyacetic acid (2,4-D). Although weak effects were seen with the native auxin IAA (Figure S1 available online), a spectrum of phenotypes of lesser-to-greater severity was observed in treatments with NAA and 2,4-D and was classified into five phenotypic classes, classes I–V (Figures 1A and S1). An increased frequency of more-severe phenotypes correlated with increasing auxin concentrations (Figure S1C). When grown on lower auxin concentrations (e.g., 100 nM NAA, 1 μM 2,4-D), class I and class II shoots were prevalent. Class I shoots appeared similar to controls, but the zone of rhizoid emergence was displaced apically, as in previous reports [47, 48, 49]. Class II shoots (seen in 2,4-D treatments) were elongated and had more leaves than controls (Figures 1A, 1C, S1A, and S1D). Class III shoots were stunted, producing fewer leaves than untreated controls (Figures 1A, 1B, and S1D), and leaves were narrow with fewer, longer cells than untreated controls (Figures 1C, S1B, and S1D). In class IV shoots, leaf outgrowth was suppressed, and gametophores comprised a raspberry-like dome of cells above a zone of rhizoid emergence (Figure 1A). Confocal microscopy revealed a spiral of successively larger leaf progenitor cells emanating from the apical cell, thus demonstrating its continued activity (Figure 1B). The strongest effect of auxin was revealed in class V shoots, which lost apical cell function. Shoots terminated with irregularly shaped cells, or rhizoids, consistent with previous reports [47, 49] (Figure 1B). These data suggest that accumulation of auxin in shoots triggers diverse developmental effects at different threshold levels. Notably, auxin accumulation causes defects in meristem function, leaf initiation, and oriented leaf growth.

### Treatment with Auxin Transport Inhibitors Phenocopies Auxin Treatment

**Figure 2:**
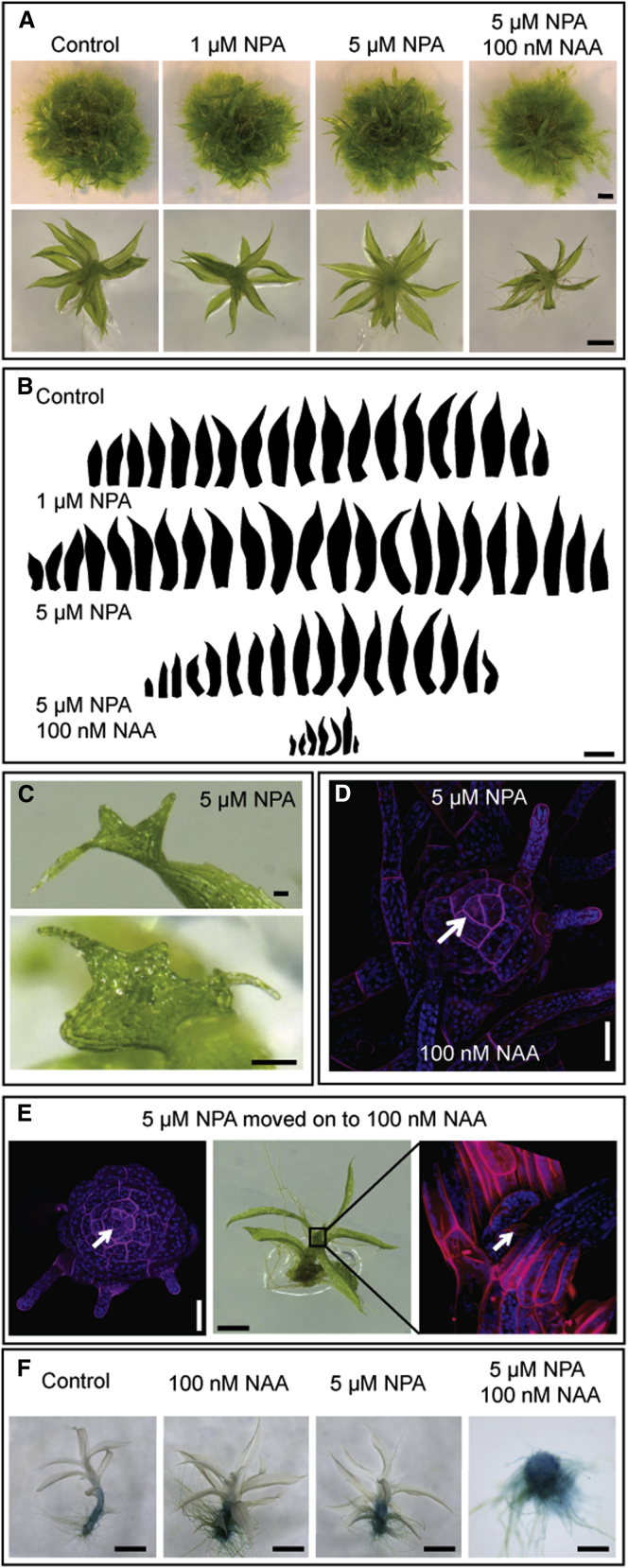
Pharmacological Polar Auxin Transport Inhibition Perturbs Leaf Development and Can Cause Meristem Arrest Plants were grown on BCD + AT medium supplemented with auxin and transport inhibitors for 3 weeks in continuous light at 23°C. (A) NPA treatment caused class I or II shoot defects, but used in combination with 100 nM NAA, it caused class III and IV defects. Scale bars represent 1 mm by row. (B) Leaf series show that 1 μM NPA caused an increase in leaf number and size relative to untreated controls. 5 μM NPA mildly inhibits leaf initiation and development, and addition of 100 nM NAA strengthens the inhibition. The scale bar represents 1 mm. (C) Treatment with 5 μM NPA caused mild perturbations to leaf development. Scale bars represent 100μm. (D) Treatment with 5 μM NPA and 100 nM NAA generated class IV shoots. The scale bar represents 50 μM. (E) If 100 nM NAA was added to plants treated with 5 μM NPA after 2 weeks, shoots that had already initiated arrested, revealing the apical cell (arrow). The scale bar represents 0.5 mm. (F) Gametophores grown for 3 weeks on control medium and medium supplemented with 100 nM NAA or medium supplemented with 5 μM NPA, or both, were stained for β-glucuronidase activity.

By analogy to flowering plants, we hypothesized that gametophore development is normally driven by changes in the auxin distribution within tissues, which was disrupted by adding exogenous auxin. We reasoned that such changes might occur by a conserved transport-dependent mechanism. To test this hypothesis, we analyzed the effect on gametophore development of the compounds 1-*N*-naphthylphthalamic acid (NPA) and naringenen (Nar), which are potent PATIs in angiosperms. Treatment with NPA caused mild developmental defects in leaves (Figure 2C), and both inhibitors had a similar effect to treatments with 2,4-D, which first promoted and then mildly suppressed leaf development (Figures 2A and 2B; Figure S2 in comparison to Figure 1; Figure S1D). However, class III–V phenotypes were not observed. Although the concentrations of NPA used here strongly inhibit auxin transport in *Arabidopsis*, the effect of PATIs is not well characterized in mosses, and we reasoned that our treatments might only partially inhibit auxin transport. We hypothesized that such partial inhibition might result in relatively mild phenotypes but might sensitize colonies to the addition of exogenous auxin. To test this hypothesis, we treated colonies with 5 μM NPA or Nar together with 100 nM NAA, which by itself only induces class I defects. These treatments gave rise to colonies with few visible gametophores that had class II and III defects (Figures 2A, 2B, S2B, and S2C): further investigation also revealed a number of class IV and V gametophores (Figures 2D and S2B). This response is similar to responses to higher concentrations of auxin applied alone, suggesting that transport normally relieves the effect of applying exogenous auxins.

### Treatment with Auxin Transport Inhibitors Can Collapse Leaf Development and Meristem Function

The severity of class IV and V responses to auxin made it difficult to determine which aspects of development are disrupted. We therefore varied this treatment by allowing plants to form normal shoots while growing on 5 μM NPA for 2 weeks before adding 100 nM NAA. During the 2 weeks following auxin addition, gametophores underwent progressive developmental arrest. Recently initiated leaves toward the apex became shorter and more slender before initiation ceased, and the apical cell was exposed (Figure 2E). In conjunction with auxin treatments, which promoted or suppressed leaf initiation (Figure S1D), these data suggest that an appropriate auxin level is required for apical cell function and is attained by transport out of the apex.

### Treatment with Auxin and Transport Inhibitors Alters the Distribution of a Marker for Auxin Response in *Physcomitrella*

The treatments with auxin and auxin transport inhibitors above suggest that the normal auxin distribution in moss gametophores is transport dependent. To evaluate this hypothesis, we analyzed the staining distribution pattern of an auxin-responsive GH3:GUS reporter [50] in untreated and pharmacologically treated plants (Figure 2F). As in previous reports [32, 50, 51, 52, 53, 54], untreated plants accumulated staining at the base of the shoot and in punctuated maxima at points of rhizoid initiation up the shoot. No staining was reproducibly detected in leaves. Treatment with 100 nM NAA increased the density of basal rhizoids and elevated the GUS staining intensity, a response that was phenocopied by treatment with 5 μM NPA. Plants that were grown on 5 μM NPA and 100 nM NAA and had class IV shoot defects accumulated stain at the shoot apex, supporting the inference that auxin transport maintains auxin levels at the apex to regulate its activity.

### *Physcomitrella* PINs Are Plasma Membrane Localized

**Figure 3:**
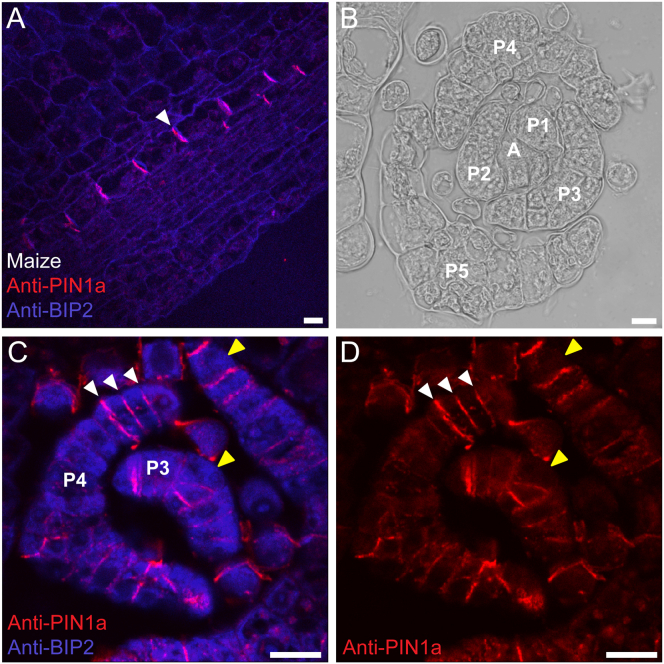
*Physcomitrella* PINs Are Plasma Membrane Targeted (A) Maize anti-PIN1a antibodies detected a strong polar signal at the plasma membrane in developing maize leaves (arrowhead). The scale bar represents 17.5 μm. (B) *Physcomitrella* leaves initiate in a spiral around the apical cell, and cell differentiation becomes apparent after P5. The scale bar represents 15 μm. (C and D) Immunolocalization in *Physcomitrella* leaves showing that anti-BIP2 (blue) and anti-PIN (red) signals do not colocalize and that the PIN signal forms a transverse banding pattern across the youngest leaf primordia around the apex (white arrows). No signal was detected at the outer cell faces (yellow arrows). Scale bars represent 15 μm.

On the basis of the data above, we reasoned that the auxin distribution in gametophore apices and leaves might be PIN regulated. We therefore used an immunolocalization approach with transverse sections just above the apex to determine where *Physcomitrella* PINs localize (Figure 3B). We used antibodies raised in guinea pigs against residues 264–413 or 264–411 of maize PIN1-like variants PIN1a and PIN1b, respectively, and, as expected on the basis of published work [55], found that both antibodies gave strong polar plasma membrane-targeted signal in maize leaf sections used as a positive control (Figures 3A and S3). We used an antibody against an abundant ER-targeted protein, BIP2, as a control to test for ER colocalization. In our moss experiments, we found that the BIP2 signal (blue) localized broadly across the undifferentiated leaf tissues of P1–P5 (Figure 3C). In contrast, the PIN signal (red) was restricted mainly to narrow bands spanning the adaxial-abaxial leaf axis at the junctions between cells and did not colocalize with the BIP2 signal (Figures 3C and 3D). We also detected signal on the internal faces of cells around the presumptive midvein, but signal at the outermost cell edges was absent. Thus, *Physcomitrella* PINs are plasma membrane targeted, can polarize, and localize in tissues that are responsive to disruption of auxin levels.

### *Physcomitrella pin* Mutants Phenocopy Plants Treated with Auxin or Auxin Transport Inhibitors

**Figure 4:**
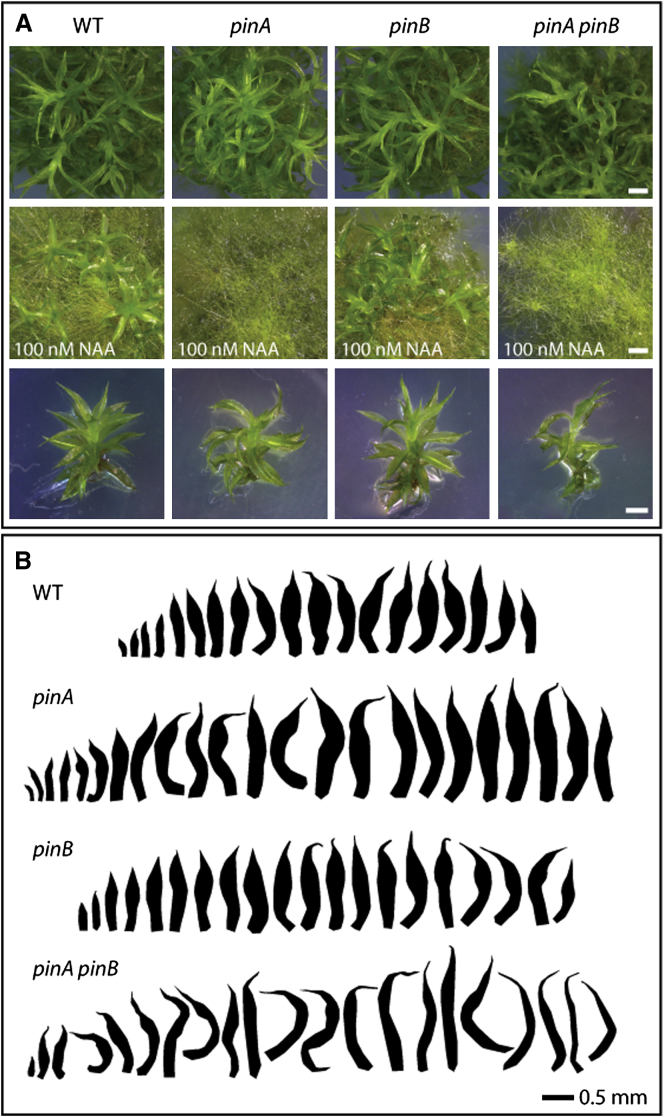
*Physcomitrella* PIN Proteins Regulate Leaf Initiation and Development Plants were grown on BCD + AT medium for 3 weeks in continuous light at 23°C. (A) Whereas *pinA* and *pinB* mutants are not easily distinguished from WT, *pinA pinB* mutants have class II shoot defects. *pinA* mutants and *pinA pinB* mutants are sensitized to NAA. The scale bar represents 1 mm by row. (B) Leaf series show subtle differences in leaf shape and size between WT and single mutants, whereas *pinA pinB* mutants have conspicuously irregularly shaped leaves that are longer and thinner than WT.

*Physcomitrella* PINs A–C are canonical and share many sequence motifs with *Arabidopsis* PIN1 in the central intracellular loop, whereas PIND is highly divergent [45], and *PINA* and *PINB*, but not *PINC*, were strongly expressed in gametophores (Figures S4A and S4B). Therefore, to analyze PIN function in *Physcomitrella*, we engineered targeted disruptants for *PINA* and *PINB* by homologous recombination [56] (Figures S4C–S4E). Several lines with the same phenotypes were recovered for each insertion, suggesting that mutant phenotypes were caused by lesions in targeted loci (Figure S4F). RT-PCR showed that disrupted *PINA* and *PINB* transcripts were present at low levels in *pinA*, *pinB*, and *pinA pinB* double mutants (Figures S4G and S4H), suggesting that the mutants may not be null. *pinA* and *pinB* single mutant shoots were not obviously different from wild-type (WT) (Figures 4A and 4B), but quantitative analysis showed that *pinB* gametophores were longer than WT (Figure S5). Double disruptants had class II shoot defects and defects in oriented leaf growth and cell division (Figures 4A and S5). *pinA pinB* double mutants therefore resemble plants treated with auxin (Figure S1), suggesting that they accumulate auxin as a result of a deficiency in auxin transport.

### *Physcomitrella pin* Mutants Are Hypersensitive to Auxin

The *pinA pinB* double mutant phenotype comprises class II defects, but more-severe defects were not observed. We reasoned that this may be due to residual PINC activity or residual activity in other components of the auxin transport pathway, such as PGP or ABC transporters [57]. We also reasoned that if we had reduced the auxin transport capacity, mutants would be more sensitive to exogenous auxin treatment than WT plants. To test this hypothesis, we grew mutants on 100 nM NAA for 4 weeks. In *pinA* and *pinA pinB* mutants, this treatment generated gametophores with class III–V phenotypes (Figure 4A), phenocopying the effect of NAA and NPA cotreatment (Figure 2A). Our results suggest that PINA and PINB act redundantly to remove auxin from the apex and initiating leaves, allowing normal development to proceed. As shoot development is strongly affected in *pinA* single mutants treated with 100 nM NAA, but not in *pinB* mutants, we postulate that PINA plays the dominant role (Figure S4B). These data support the hypothesis that the apical auxin distribution in *Physcomitrella* regulates gametophore architecture and is modulated by PIN proteins.

### A Marker for Auxin Response Is Redistributed in *Physcomitrella pin* Mutants

**Figure 5:**
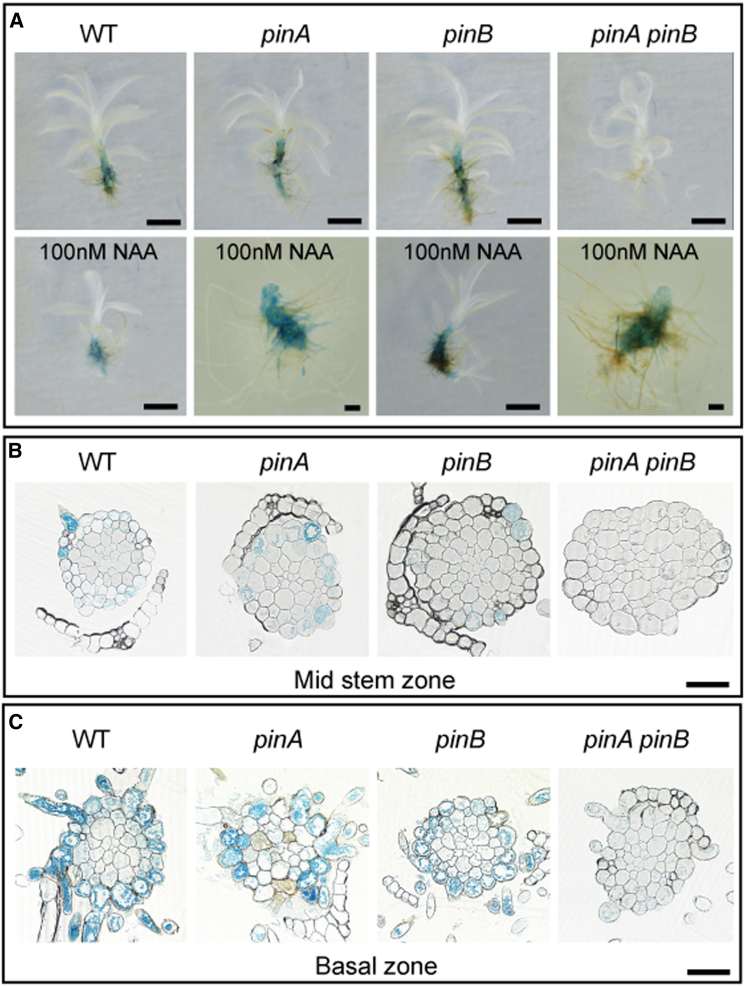
A GH3:GUS Reporter Is Redistributed in *pinA pinB* Mutant Shoots (A) GH3:GUS expression in the WT, *pinA*, *pinB*, and *pinA pinB* lines was assessed after 3 weeks of growth on control medium (top row) or medium supplemented with 100 nM NAA (bottom row). Gametophores were extracted and then stained for β-glucuronidase activity for 30 min. Scale bars (long) represent 1 mm; scale bars (short) represent 100 μm. (B) Transverse sections through the midstem region showed a patterned distribution of epidermal staining in WT, *pinA* and *pinB* plants. In *pinA pinB* mutants, the staining intensity was much reduced. The scale bar (long) represents 1 mm (C) Transverse sections through the basal region showed strong, evenly distributed epidermal staining in WT, *pinA* and *pinB* plants. In *pinA pinB* mutants, the staining intensity was much reduced or absent. The scale bar (long) represents 1 mm

To further test the hypothesis that PIN proteins modulate the auxin distribution in *Physcomitrella*, we analyzed the staining distribution pattern of the GH3:GUS reporter [50] in WT and mutant plants (Figure 5A). In *pinA* and *pinB* single mutant shoots, staining was slightly stronger than in WT and displaced up the stem. In contrast, the staining intensity in *pinA pinB* mutants was strongly reduced with respect to WT and single mutants and, where present, was localized to the middle portion of the stem. Gametophores with the most-severe leaf phenotypes had the least signal and very few rhizoids initiated; no basal zone of rhizoid emergence was apparent (Figures 5A–5C). Transverse sections taken through the base and midstem region confirmed this inference, indicating a difference in the apical-basal auxin level and distribution as the main defect (Figures 5B and 5C). To test whether auxin-inducible phenotypic alterations to shoot development (Figure 3A) corresponded to an altered auxin response distribution, plants were grown on 100 nM NAA before staining. Whereas gametophores with a class I–III response showed only an upregulation in signal intensity, *pinA* and *pinA pinB* mutants with class IV and V phenotypes accumulated staining toward or at the apex (Figure 5A). These data support the hypothesis that PIN proteins modulate the auxin distribution in gametophores.

### *Physcomitrella pin* Mutants Have Disrupted Tropic Responses

**Figure 6:**
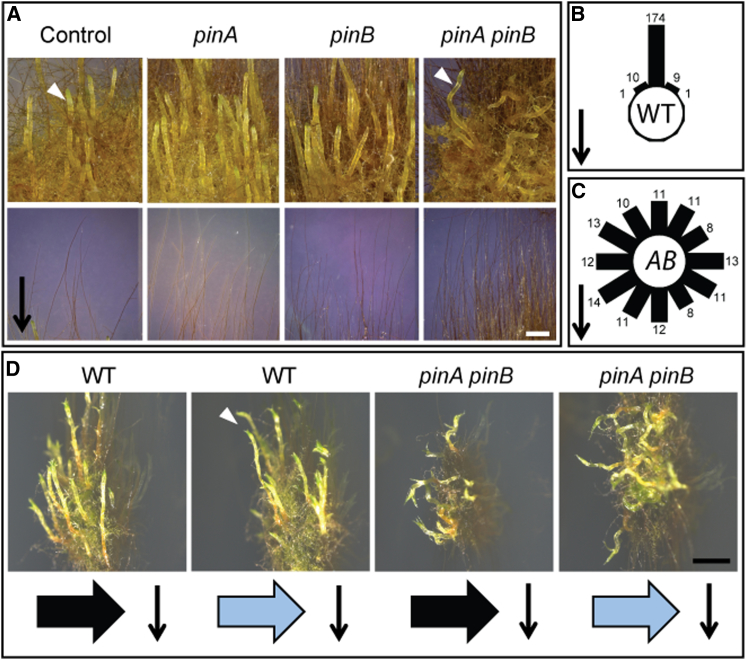
PIN Proteins Mediate *Physcomitrella* Shoot Tropism Plants were grown on BCD + AT medium for 3 weeks horizontally in continuous light at 23°C before plates were wrapped in foil, oriented vertically, and allowed to grow for 2 more weeks. (A) Whereas filaments reoriented away from the new gravity vector in all genotypes, shoot gravitopism was abolished in *pinA pinB* mutants. The scale bar represents 500 μm. (B and C) For WT (B) and *pinA pinB* (C), the response was quantified by counting the number of shoot tips in 30° sectors relative to the gravity vector. (D) Dark-grown colonies of WT and *pinA pinB* mutant plants were exposed to unidirectional blue light (blue arrows) for 24 hr to assess the phototropic response of etiolated gametophores. Whereas control shoots were kept in the dark and maintained their previous growth vectors (vertical arrows), WT shoots reoriented toward the light source (arrowhead). *pinA pinB* mutant shoots showed no obvious reorientation. The scale bar represents 500 μm.

In angiosperms, PIN-mediated polar auxin transport drives phototropic and gravitropic responses in shoots and roots [58, 59]. *Physcomitrella* filaments and gametophores have strong negative gravitropism when grown in the dark [60]. Interestingly, moss mutants defective in filament gravitropism are not defective in shoot gravitropism, suggesting that two distinct tropism pathways may operate [60]. To assess a putative role for PIN-mediated auxin transport in gravitropism, we grew WT, single and double *pin* mutants for 2 weeks in the light and then grew them vertically in the dark on sucrose supplemented medium (0.5% w/v) for a further 2 weeks. In WT plants, this treatment induced a strong negative gravitropic response in both filaments and gametophores (Figures 6A–6C). Whereas *pinA* and *pinB* single mutants showed a normal gravitropic response, the *pinA pinB* double mutant had agravitropic gametophores. This result was phenocopied by treatment with 2,4-D (data not shown). To assess a putative role in phototropism, we grew plants as above but then exposed them to a unidirectional blue light stimulus for 24 hr. Whereas the tips of WT gametophores showed a clear reorientation toward the light stimulus (Figure 6D), *pinA pinB* colonies subjected to the same light stimulus continued to grow in a disoriented manner, showing no clear tropic growth toward the light stimulus (Figure 6D). These data suggest conservation of PIN-dependent, auxin transport-driven gravitropism and phototropism pathways between mosses and angiosperms and again highlight the importance of auxin transport-driven processes in *Physcomitrella* gametophore development.

### *Physcomitrella pin* Mutants Have Disrupted Sporophyte Development

**Figure 7:**
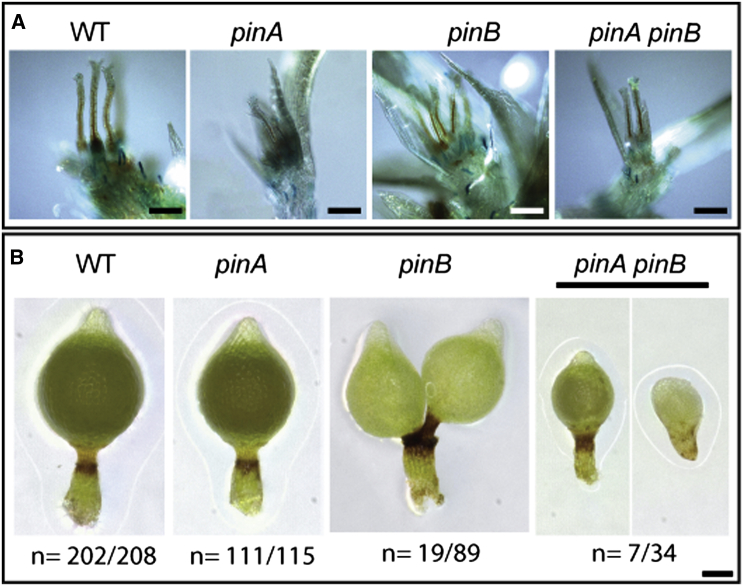
PIN Proteins Regulate *Physcomitrella* Sporophyte Development Plants were grown on peat plugs in continuous light for 6 weeks at 23°C before transfer to a short-day regime at 16°C. All visible sporophytes were dissected out of gametophores after a further 4 weeks and photographed using a Keyence VHX-1000 microscope. (A) Gametangium development appeared normal. Scale bars represent 75 μM. (B) Gross phenotypic perturbations were rare in WT or *pinA* lines but occurred with variable penetrance in *pinB* and *pinA pinB* lines. The scale bar represents 100 μM.

For reasons outlined in the introduction, this study has principally targeted recent controversy surrounding the roles of auxin transport in *Physcomitrella* gametophore development. However, as auxin transport has previously been detected in moss sporophytes and application of transport inhibitors perturbs sporophyte development [32], we also tested the hypothesis that PIN-mediated auxin transport regulates sporophyte development. We detected sporophytic expression of *PINA* and *PINB* (Figure S4B) and grew WT and *pin* mutant sporophytes to evaluate their phenotypes. Cultures were grown on four peat plugs in continuous light at 23°C for 6 weeks before transfer to a short-day 16°C regime for induction, and all the sporophytes present were harvested 4 weeks after induction. Whereas gametangia appeared normal (Figure 7A), PINA and PINB contributed synergistcially to fertility and development (Figures 7B and S6). Sporophytic defects were detected with variable penetrance: a low proportion (6 out of 208) on our GH3:GUS WT line had duplicated sporangia or dead sporophytes. Whereas *pinA* mutants had no obvious defects (1 out of 115 had duplicated sporangia; 3 out of 115 had an enlarged sporangium), a significant proportion of *pinB* mutants had duplicated sporangia (19 out of 89; 6 out of 89 were dead or had other defects), and around half of *pinA pinB* mutants had severe, sometimes lethal, developmental defects (5 out of 34 had duplicated sporangia; 7 out of 34 were dead or had other defects). The results suggest that PIN-mediated auxin transport regulates sporophytic shoot development, with a stronger contribution from *PINB* than from *PINA*.

## Discussion

### *Physcomitrella* PINs Can Polarize at the Plasma Membrane

On the basis of heterologous gene expression assays in tobacco, previous work suggested that *Physcomitrella* PINs A and D localize at the ER and cytosol, respectively, and land plant PINs were therefore postulated to have an ancestral role in regulating intracellular auxin homeostasis rather than intercellular transport [34, 35]. However, we have recently shown that *Physcomitrella* PINA–PINC are canonical, sharing sequence motifs that are required for plasma membrane targeting with *Arabidopsis* canonical PINs [45]. Our work suggested that canonical PINs are one ancestral type within the land plants and that *Physcomitrella* PINs A–C should have a capacity for plasma membrane targeting [45]. Using immunolocalization, we have found that *Physcomitrella* PINs A–C can indeed target the plasma membrane; we did not detect signal elsewhere in cells, and we did not detect signal colocalizing with an ER marker.

*Physcomitrella* PIN localization usually formed a conspicuous banding pattern traversing the adaxial-abaxial leaf axis, where two cells contact one another (Figures 3 and S3). Where leaves were thickened around the midvein, we also detected signal on the cell faces that were in contact with other cells, but the outermost cell faces were usually free from signal. Although we cannot rule out the possibility that each neighboring cell contributes to the high signal intensity at cell junctions, in our view, the localization is polarized. As auxin-treated gametophores and *pinA pinB* mutants have around half the number of cells in the mediolateral leaf axis than normal and the mediolateral leaf axis is elaborated by asymmetric cell divisions [61], a polar localization pattern perpendicular to the mediolateral axis is consistent with a role for PINA and PINB in promoting asymmetric cell division. These results suggest a role for canonical *Physcomitrella* PINs in intercellular polar auxin transport in leaf development.

### PIN-Mediated Auxin Transport Drives Meristem Function and Leaf Development

Recent work was unable to detect polar auxin transport in gametophytic moss shoots, and no effect of treatment with transport inhibitors was observed, leading to the conclusion that auxin transport does not contribute to gametophore development [32, 33]. We were also unable to detect long-range polar auxin transport using radio-labeled IAA (data not shown). The discrepancy between the results that we obtained with NPA and previously published results arises from a difference in experimental approach. Whereas previous experiments immersed fully grown shoots in 50 μM NPA [32, 33], we grew colonies on NPA, exposing shoots to transport inhibition from the earliest developmental stages, and cotreatment with low auxin concentrations was needed to see strong developmental effects (Figure 2). We found that treatment of WT gametophores with NPA disrupted extension of proximodistal and mediolateral axes of leaf development and disrupted meristem function. The effects observed were similar to treatments with high concentrations of auxin or treatments of *pinA* mutants with low concentrations of auxin. Again, these results support a role for PIN-mediated auxin transport in the asymmetric cell divisions that drive leaf development and meristem function [61]. Consistent with PIN localization patterns, we hypothesize that auxin transport in moss gametophores occurs in a localized manner, to remove auxin from the leaves and meristem without detectable long-distance flux [62]. It is also possible that *Physcomitrella* PINs distribute auxin principally in the epidermis and, therefore, that the overall levels of transport involved are low.

### PIN-Regulated Shoot Development Is a Deep Homology of Stomatophytes

Collectively, our data show that auxin transport regulates a suite of characteristics in *Physcomitrella* gametophore development that are similar to the developmental characteristics that are PIN regulated in angiosperm sporophytes, and our inferences are supported by data from Viaene et al. [63], published in this issue of *Current Biology*. PIN-mediated auxin transport in *Physcomitrella* regulates intrinsic developmental processes, such as asymmetric cell division, growth, meristem function, and leaf development, and dynamic responses to the environment, such as shoot tropisms. In conjunction with recently published results showing that charophytes have a capacity for long-range polar auxin transport [41], the regulation of these aspects of gametophore development in *Physcomitrella* raises the possibility that auxin transport could be a core mechanism for plant development that was recruited from the gametophyte to the sporophyte during land plant evolution. Alternatively, the roles of PIN-mediated auxin transport could have evolved convergently in moss gametophores. In either case, the recruitment of PIN-mediated auxin transport to regulate gametophore development is a clear instance of deep homology within the stomatophytes and the first that affects such general developmental programs.

### PIN-Mediated Auxin Transport Is a Conserved Regulator of Sporophyte Development

Work in *Selaginella* has shown that the roles of polar auxin transport in regulating apical meristem function and shoot branching are conserved within the vascular plants [28, 29, 30, 31]. Previous work in mosses has shown that bulk polar auxin transport in sporophytes can be disrupted by NPA treatment, causing multiple sporangia to form [32, 33]. Our data also support the notion that sporophyte development in *Physcomitrella* is regulated by polar auxin transport [32, 33]. We have demonstrated that *PINA* and *PINB* are expressed in sporophytes and contribute synergistically to fertility and development (Figure 7); PIN-mediated auxin transport is a conserved regulator of sporophyte development in stomatophytes. We note that the duplicated sporangium phenotype of *pinB* and *pinA pinB* mutants reproduces branching morphologies of early prevascular fossils, such as *Partitatheca* [13], and speculate that this phenotype could arise by an early embryonic duplication of the apical cell, or bifurcation [64, 65, 66]. PIN-mediated auxin transport is a major driver of plant architecture in flowering plants [17], and changes in meristem function underpin architectural divergence between plant groups [4, 67]. The identification of conserved roles for auxin transport in land plant meristem function opens the possibility that PIN proteins played a key role in the radiation of plant form.

## Experimental Procedures

A GH3:GUS reporter line [50] was used as the WT moss strain. Spot cultures were grown as described previously [61], and tissue for genetic analysis was prepared as in [50]. All lines were stored in the International Moss Stock Center (http://www.moss-stock-center.org; see Supplemental Information).

For immunolocalizations, tissue was grown for 4 weeks in continuous light, fixed in 3:1 methanol acetic acid, dehydrated, and embedded in PEG 1600. Eight-micrometer sections were interrogated with anti-maize PIN antibodies [55] at a 1/150 dilution and anti-BIP2 (Agrisera) at a 1/50 dilution. DyLight 594 and DyLight 405 were used as secondary antibodies at a 1/300 dilution.

*pin* disruptants were generated and screened for insertion as described in Supplemental Information.

GUS staining was carried out as elsewhere [32]. Light micrographs were compiled using a Keyence VHX-1000 series microscope with 50× and 200× objectives. Confocal imaging was undertaken as previously described [61], except for immunolocalizations; a Leica TCS 5 was used, with excitation from the Diode 405 and HeNe 594 laser lines, and emission was collected at 410–480 nm and 600–670 nm.

## Author Contributions

E.L.D., R.R., and C.J.H. conceived this study. All authors contributed to experimental design. Foundational experiments were undertaken by T.A.B., M.M.L., T.A., N.M.B., M.B., X.Y.W., C.D.W., and C.J.H., with supervision from E.L.D., R.R., and C.J.H. T.A.B. contributed Figures 6B–6D, S1C, and S2B; M.M.L. contributed Figures 5B and 5C; Y.C. contributed Figure 7B; T.A. contributed Figures S4G and S4H; R.J.D. contributed Figures S1D, S2A, and S5; E.L.D. contributed Figure S4A; C.D.W. contributed Figure S4B; X.Y.W. contributed Figure S4F; and C.J.H. contributed the remainder. T.A.B., M.M.L., T.A., R.J.D., E.L.D., R.R., and C.J.H. contributed to data analysis and interpretation. The final manuscript was drafted by C.J.H., with help from T.A.B., T.A., E.L.D., and R.R. C.J.H. handled submission. D.O. contributed anti-PIN antibodies and technical help with immunohistochemistry.

## Supplemental Information

Document S1. Supplemental Experimental Procedures and Figures S1–S6

Document S2. Article plus Supplemental Information
