# Supplementary material for: Plasma Membrane-Targeted PIN Proteins Drive Shoot Development in a Moss
Source: Curr Biol. 2014 Dec 1;24(23):2776–85. doi: 10.1016/j.cub.2014.09.054 (PMC4251699; doi:10.1016/j.cub.2014.09.054)
Supplement: Document S1. Supplemental Experimental Procedures and Figures S1–S6 [file mmc1.pdf]

Current Biology, Volume *24*

Supplemental Information

Plasma Membrane-Targeted PIN Proteins Drive Shoot Development in a Moss

Tom A. Bennett, Maureen M. Liu, Tsuyoshi Aoyama, Nicole M. Bierfreund, Marion Braun, Yoan Coudert, Ross J. Dennis, Devin O'Connor, Xiao Y. Wang, Chris D. White, Eva L. Decker, Ralf Reski, and C. Jill Harrison

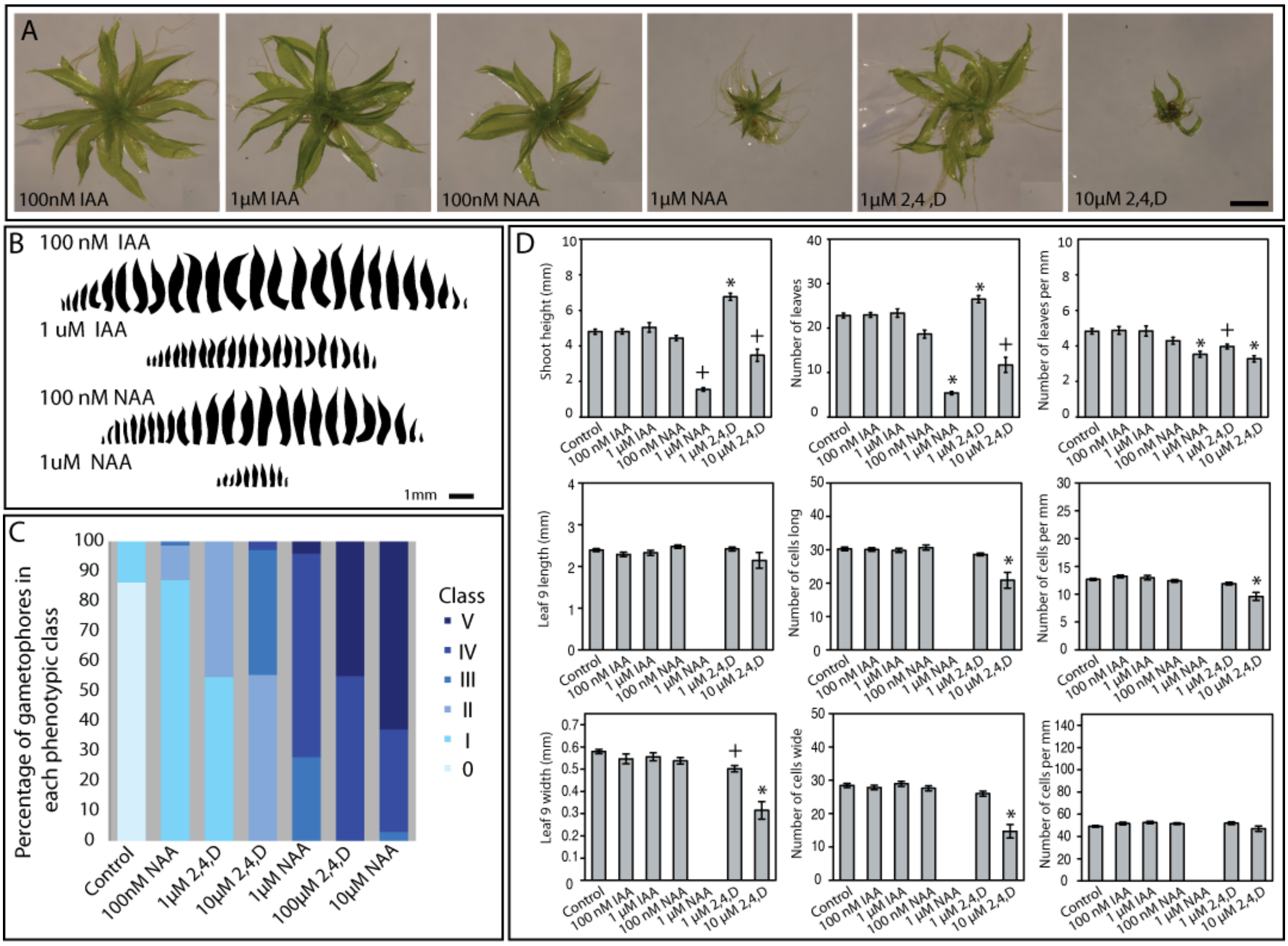

Figure S1 related to Figure 1: Different auxins have a similar developmental effects.

(a) Gametophores from plants treated with different auxins. Scale bar = 1 mm.

(b) Leaf series from plants grown on IAA and NAA. Scale bar = 1 mm.

(c) The proportion of gametophores per colony in each phenotypic class varied by treatment.

(d) Auxin accumulation at low levels promotes shoot growth and apical cell activity, but at high levels the effect is converse. Accumulation of auxin in leaves inhibits proximo-distal and medio-lateral divisions, but promotes proximo-distal expansion. Data is shown from one experimental replicate of three in which mean values and the standard error were calculated from a sample of 20 shoots. Inferences that were statistically significant in all three replicates are indicated by an asterisk, and those that were statistically significant in two replicates are indicated by a cross.

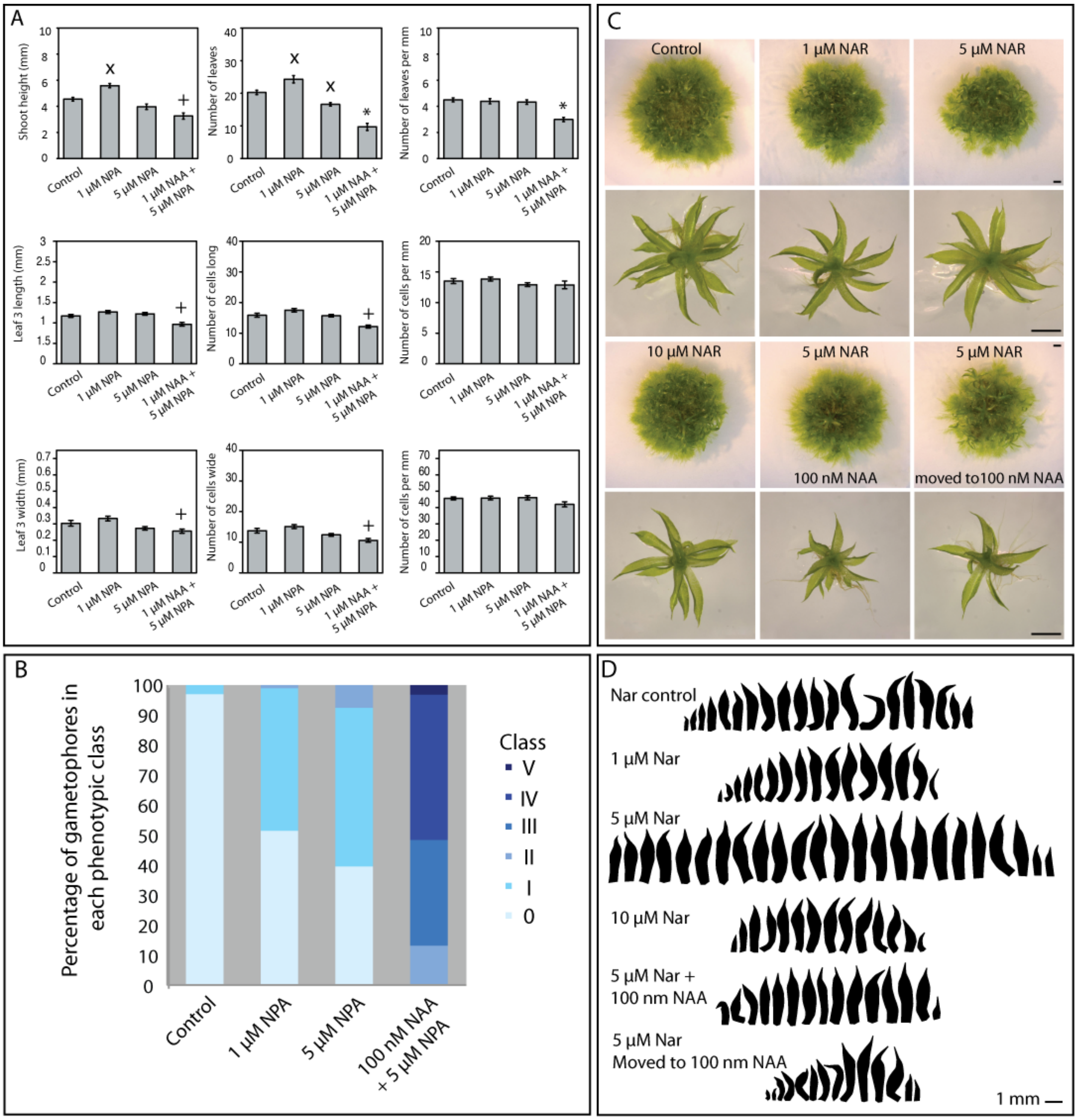

Figure S2 related to Figure 2: Effects of transport inhibition on development.
(a) Treatment with 1μM NPA phenocopies treatment with 1 μM 2,4-D and promotes shoot growth and leaf intiation, whereas 5μM NPA has a mild inhibitory effect. Addition of 100 nM NAA increases the inhibitory effect, and phenocopies 10 μM 2,4-D treatments: leaves are narrow with fewer cells by width. Data is shown from one experimental replicate of three in which mean and standard error values were calculated from a sample of 20 shoots where possible. Inferences that were statistically significant are marked by an asterisk (all three replicates), a + (two replicates), and an x (this replicate).
(b) The proportion of gametophores per colony in each phenotypic class varied by treatment.
(c) Treatment with naringenin did not strongly affect colony or gametophore development. Further addition of 100 nM NAA caused Class III defects. Scale bars = 1 mm.
(d) Whereas 5 μM naringenin (Nar) mildly promoted leaf initiation and growth, 10 μM naringenin or 5 μM naringenin with 100 nM NAA inhibited leaf initiation and growth.

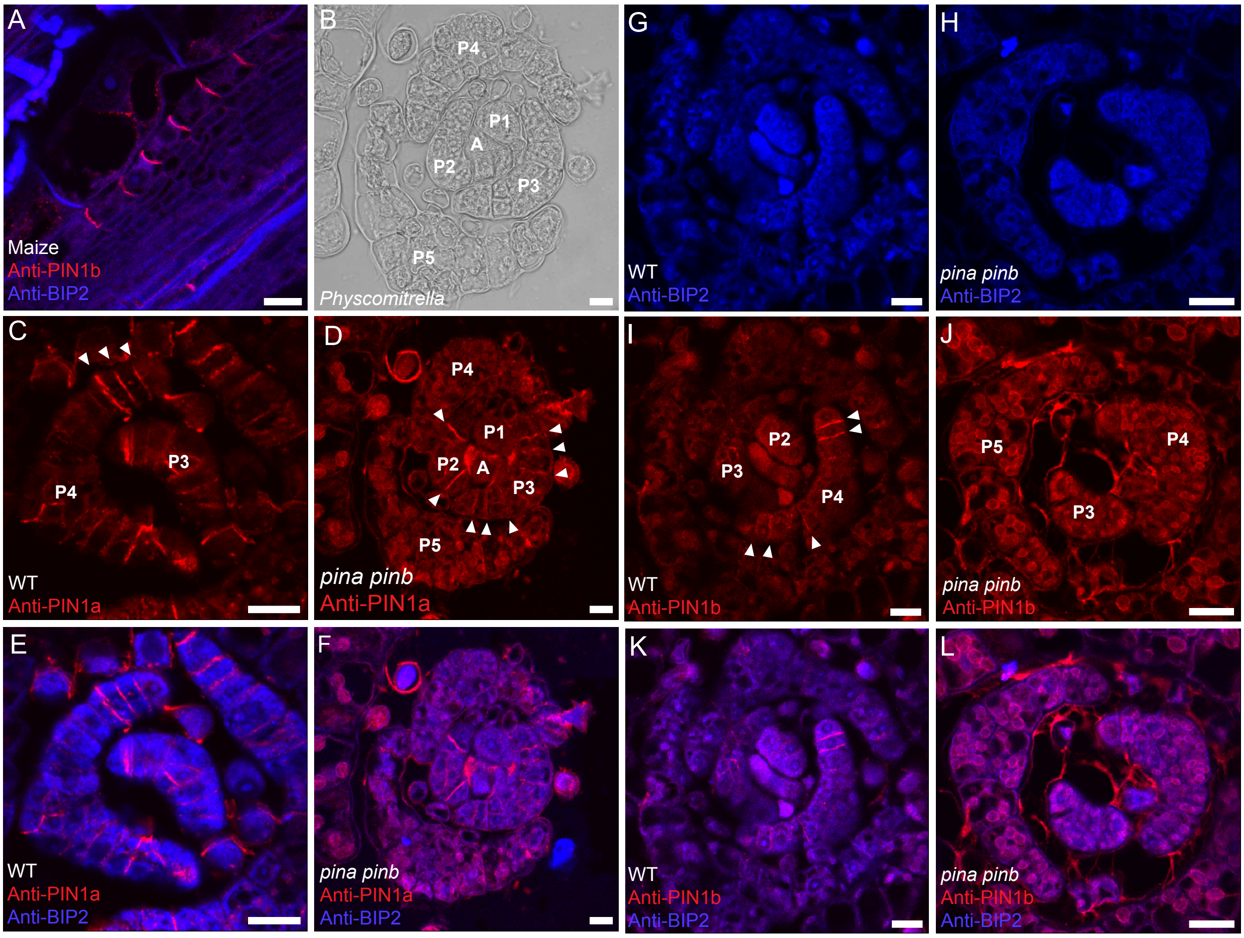

Figure S3 related to Figure 3: Immunolocalization controls.
(A) Longitudinal section showing that maize anti-PIN1b antibodies detected polar, plasma membrane targeted signal in maize leaves. Scale bar = 17.5 µm.
(B) Transverse section across a *Physcomitrella* apex showing leaves P1-P5 initiating in a spiral around the apical cell (A). Scale bar = 15 µm.
(C-F) Sections interrogated with maize anti-PIN1a antibodies in WT and *pinA pinB* mutants respectively. Note that the *pinA pinB* mutant is not null (see Figures 5 and S4), and that signal detected therefore likely reflects residual PIN accumulation. Scale bars = 15 µm.
(G-L) Immunolocalizations with the anti-PIN1b antibody showing that a similar signal distribution is detected in *Physcomitrella* with PIN1a and PIN1b antibodies. However, the signal detected is absent in *pinA pinB* mutants, suggesting that the maize anti-PIN1b antibody specifically targets PIN proteins in *Physcomitrella*. Scale bars = 15 µm.

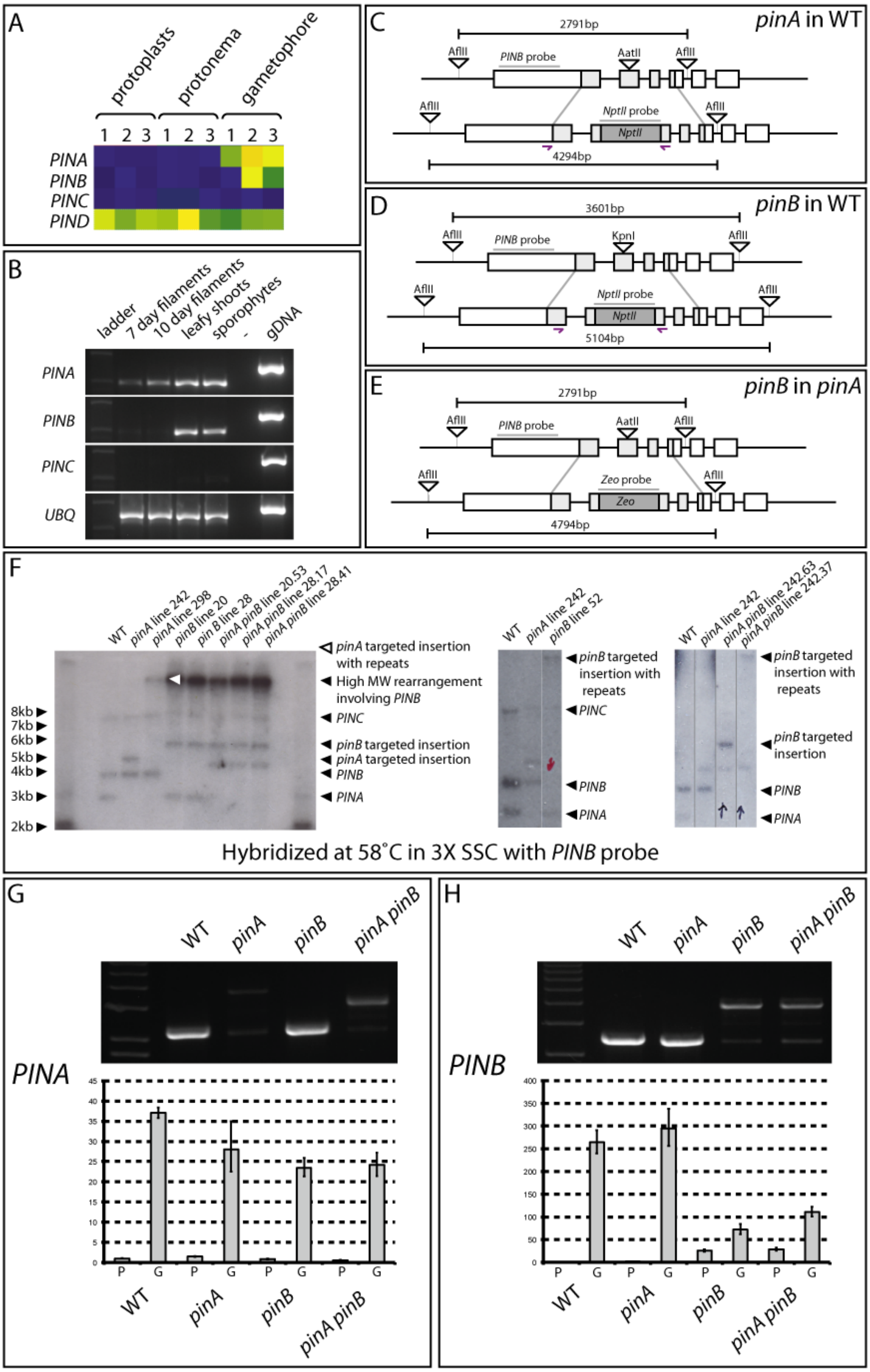

Figure S4 related to Figure 4: Expression patterns, insertion strategy and genetic analysis of *PIN* loci in WT and *pin* mutant lines.
(A) Tile display of the expression pattern of *Physcomitrella PIN* orthologues detected on a Combimatrix whole genome array.
(B) RT-PCR detected expression of *PINA* in filaments, leafy shoots and sporophytes. *PINB* was expressed strongly in leafy shoots and sporophytes. *PINC* was expressed weakly in leafy shoots and sporophytes.
(C-E) Insertion strategy for *PIN* disruption and the location of probe and primers (purple arrows) used in genetic analyses.
(F) Southern analysis with a *PINB* or resistance casette probes (not shown) confirmed targeted insertion of disruption casettes at the *PINA* and *PINB* loci in wild-type and *pinA* mutant plants.
(G, H) RT-PCR and Q-PCR showed disrupted *PINA* and *PINB* expression in *pinA*, *pinB* and *pinA pinB* mutants. Graphs show expression levels of *PINA* or *PINB* in gametophore (G) tissue relative to protonemal tissue (P) as fold change. In both *pinA* and *pinB* mutants we found two PCR amplicons; the long transcript had a c.1.7kb insertion deriving from the resistance casette at amino acid position 524 (*pinA*) or 537 (*pinB*). The short transcript from the *pinA* mutant had a 5bp deletion which introduced a stop codon at amino acid position 530, and the short transcript from the *pinB* mutant had a 9bp deletion corresponding to amino acids 543-546. All of these disruptions are in the intracellular loop region, and whilst the short *pinB* transcript could potentially generate a functional protein, it was expressed at very low levels (Fig. S4H).

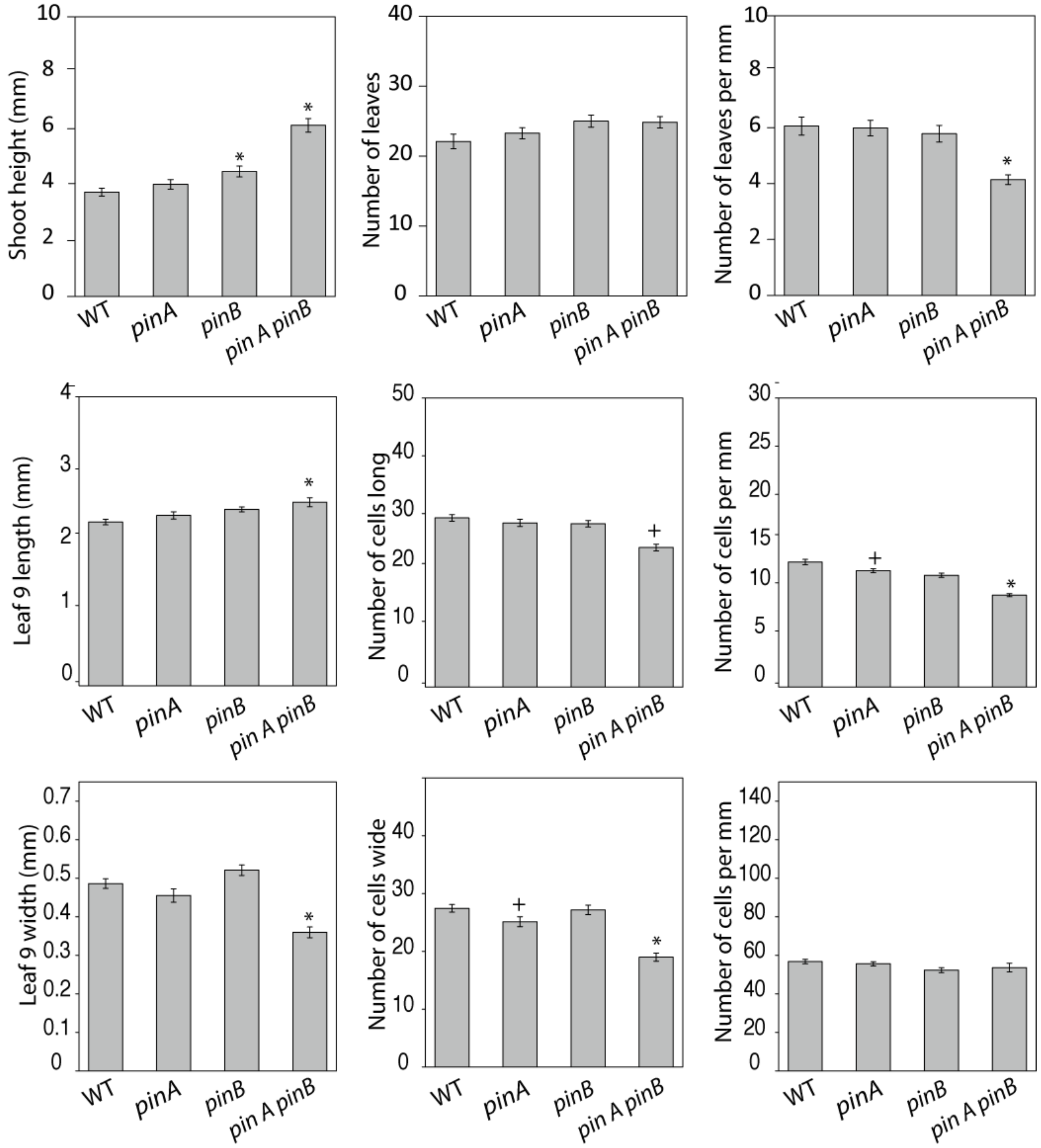

Figure S5 related to Figure 4: *pinA pinB* mutants have longer shoots with more leaves but fewer leaves per unit length than WT. *pinA pinB* mutants have longer leaves with fewer, longer cells than WT. Leaves are narrower with fewer cells by width than WT.

Data shown is from one experimental replicate of three in which mean values and the standard error were calculated from a sample of 20 shoots. Inferences that were statistically significant in all three replicates are indicates by an asterisk, and those that were statistically significant in two replicates are indicated by a cross.

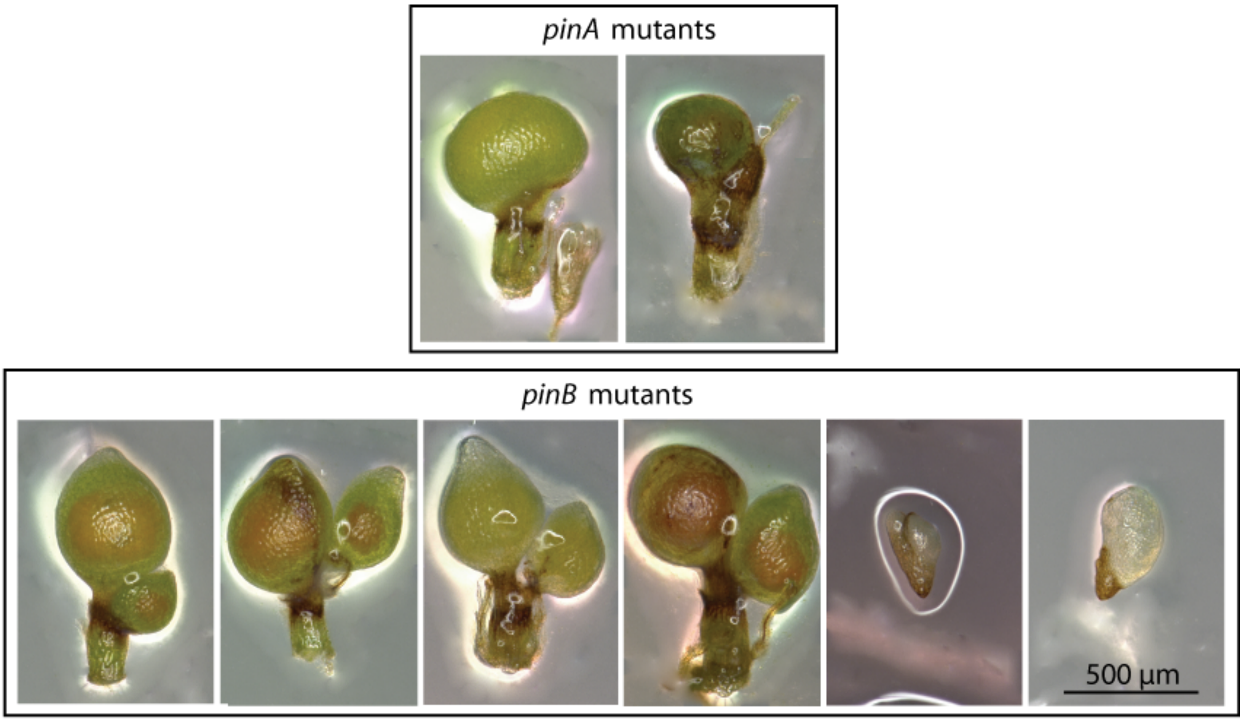

Figure S6 related to Figure 7: *pin* mutant phenotypes were variable.

Supplemental Methods.

Construct generation for insertion lines

To generate *pinA* and *pinB* disruption constructs, genomic DNA fragments corresponding to respective genes were PCR amplified using the primers Pin37 and Pin36 (*PINA*) and Pin-2C and Pin-2D (*PINB*) and cloned into pCR®4-TOPO® (Invitrogen). The *PINA* genomic fragment was disrupted by insertion of the nptII selection casette in an internal AatI restriction site (Fig. S4a). *PinB* disruptants were made by inserting the nptII casette into an internal KpnI restriction site (Fig. S4b), and double disruptants were made by replacing the nptII casette in *pinA* constructs with a zeomycin resistance casette obtained from pRT101-zeo[S1], and re-transformation into *pinB* mutant backgrounds (Fig. S4c). Prior to transformation, the insertion cassettes were released from the TOPO backbone via EcoRI digest.

Screening

Stable disruptant lines were screened for insertion as described in Fig. S4. Two *pinA* disruptants, two *pinB* disruptants, and three double disruptants had targeted insertion and loss of expression, and mutant phenotypes were shared by gene disrupted (not shown). These lines have been stored in the International Moss Stock Center as follows: GH3:GUS: IMSC#40283, *pinA* line 242: IMSC#40474, *pinA* line 298: IMSC#40475, *pinB* line 20: IMSC#40477, *pinB* line 28: IMSC#40478, *pinB* line 52: IMSC#40752, *pinA pinB* line 20.53: IMSC#40583, *pinA pinB* line 28.17: IMSC#40580, *pinA pinB* line 28.41: IMSC#40582, *pinA pinB* line 242.63: IMSC#40753, *pinA pinB* line 242.37: IMSC#40754.

Expression analyses

Evaluation of *PIN* expression by array was undertaken as described elsewhere[S2] using a cut off value of 10^4 for expression. To evaluate expression by RT-PCR, 1 µg RNA was extracted with a Qiagen RNeasy™ plant mini kit, DNAse treated then converted to cDNA with superscript III. PCR in WT (Figure S4b) was carried out using primers 5-10 listed below with an initial denaturation step of 2 minutes at 94 degrees and 30 cycles of 15 s at 94˚C, 15 s at 61˚C and 60 s at 72˚C. A final 5 minute 72˚C step was also used. PCR to screen mutants (Figure S4g, h) was carried out using primers 13-15 listed below with an initial denaturation step of 2 minutes at 98 degrees and 30 cycles of 10 s at 95˚C, 30 s at 60˚C and 2 min at 72˚C.

For Q-PCR, cDNA was synthesized using the QuantiTect Reverse Transcription Kit (QIAGEN). Quantitative RT-PCR analysis was performed using QuantiTect SYBR Green PCR Kit (QIAGEN) and CFX96 PCR machine (Bio-Rad). The following primer pairs were used: for PINA, PINA_qPCR_F1 and PINA_qPCR_R1; for PINB, PINB_qPCR_F1 and PINB_qPCR_R1, UBI, UBI-F-QPCR and UBI-R-QPCR. An initial denaturation step of 15 minutes at 94 degrees and 40 cycles of 15 s at 95˚C, 30 s at 60˚C and 30 s at 72˚C were used.

Primers used

1. Pin37: CCAGGAAGCCAAACAGCCATC
2. Pin36: GGCTGCAGCAAATACAGCTGG

3. Pin-2C: CTCCACGGGCTTCTCAAATC
4. Pin-2D: CCCAATCCCATGAACAAGCC

5. PINA-RT-F: TCCAGGAAGCCAAACAGCCAT
6. PINA-RT-R: CTCTGCCAGTTTCGGTGTCAA

7. PINB-RT-F: GTCTTGTTACTCCCGGAGGTA
8. PINB-RT-R: CTTTGCTTCGTCTTCGGGTA

9. PINC-RT-F: CGATATCTCCATTAACCTCCA
10. PINC-RT-R: GACTGAACATGGCCATCCCAA

11. Ubi-F: GCCATGCAGATCTTCGTGAA
12. Ubi-R: CTACGCAGCCAAGAACCGA

13. PINA_RT_F: TTTGGAGGTTTTCGTTTTTGG
14. PINB_RT_F: GGAGATTTGGACTGCCTCAG
15. PIN_R: TCACAGACCAAGTAATATGTAGT

16. PINA_qPCR_F1: CCCGAGAATTTGTTCCTTCA
17. PINA_qPCR_R1: CACCACTTCACAGAGCCGTA

18. PINB_qPCR_F1: AATTGTTGTGTGCGGACGTA
19. PINB_qPCR_R1: TCACCGCAGTACTGAGCATC

20. UBI-F-QPCR: CGTGCGTTGTGAGTGTTTAGA
21. UBI-R-QPCR: GCAGCCAAGAACCGATAGAT

Supplementary References

S1. Parsons, J.F. Altmann, C.K. Arrenberg, A. Koprivova, A.K. Beike, C. Stemmer, G. Gorr, R. Reski, E.L. Decker (2012): Moss-based production of asialo-erythropoietin devoid of Lewis A and other plant-typical carbohydrate determinants. Plant Biotechnology Journal *10*: 851-861

S2. Wolf, L., Rizzini, L., Stracke, R., Ulm, R. and Rensing, S. (2010) The Molecular and Physiological Responses of Physcomitrella patens to Ultraviolet-B Radiation. Plant Physiology *153*: 1123-1134
