# Supplementary material for: Plasma Membrane-Targeted PIN Proteins Drive Shoot Development in a Moss
Source: Curr Biol. 2014 Dec 1;24(23):2776–85. doi: 10.1016/j.cub.2014.09.054 (PMC4251699; doi:10.1016/j.cub.2014.09.054)
Supplement: Document S2. Article plus Supplemental Information [file mmc2.pdf]

Current Biology 24, 2776–2785, December 1, 2014 ©2014 The Authors http://dx.doi.org/10.1016/j.cub.2014.09.054

Article

Plasma Membrane-Targeted PIN Proteins Drive Shoot Development in a Moss

Tom A. Bennett,[1,2,6] Maureen M. Liu,[1,6] Tsuyoshi Aoyama,[1,6] Nicole M. Bierfreund,[3] Marion Braun,[3] Yoan Coudert,[1] Ross J. Dennis,[1] Devin O'Connor,[2] Xiao Y. Wang,[1] Chris D. White,[1] Eva L. Decker,[3] Ralf Reski,[3,4,5] and C. Jill Harrison[1,*]

[1]Plant Sciences Department, University of Cambridge, Downing Street, Cambridge CB2 3EA, UK
[2]Sainsbury Laboratory, University of Cambridge, Bateman Street, Cambridge CB2 1LR, UK
[3]Faculty of Biology, University of Freiburg, Schänzlestraße 1, 79104 Freiburg, Germany
[4]BIOSS Centre for Biological Signalling Studies, 79104 Freiburg, Germany
[5]Freiburg Institute for Advanced Studies (FRIAS), 79104 Freiburg, Germany

Summary

Background: Plant body plans arise by the activity of meristematic growing tips during development and radiated independently in the gametophyte (n) and sporophyte (2n) stages of the life cycle during evolution. Although auxin and its intercellular transport by PIN family efflux carriers are primary regulators of sporophytic shoot development in flowering plants, the extent of conservation in PIN function within the land plants and the mechanisms regulating bryophyte gametophytic shoot development are largely unknown.

Results: We have found that treating gametophytic shoots of the moss *Physcomitrella patens* with exogenous auxins and auxin transport inhibitors disrupts apical function and leaf development. Two plasma membrane-targeted PIN proteins are expressed in leafy shoots, and *pin* mutants resemble plants treated with auxins or auxin transport inhibitors. PIN-mediated auxin transport regulates apical cell function, leaf initiation, leaf shape, and shoot tropisms in moss gametophytes. *pin* mutant sporophytes are sometimes branched, reproducing a phenotype only previously seen in the fossil record and in rare natural moss variants.

Conclusions: Our results show that PIN-mediated auxin transport is an ancient, conserved regulator of shoot development.

Introduction

Land plants evolved from freshwater algae with a haploid-dominant life cycle in which meiosis occurred straight after fertilization, and the colonization of land around 450 million years ago was accompanied by the innovation of a multicellular diploid body [1–4]. Complex morphologies diversified independently in both the haploid (gametophyte) and diploid (sporophyte) life cycle stages in different plant groups during evolution [4, 5]. Bryophytes comprise a basal, gametophyte-dominant grade [6–8] with widely divergent thalloid, filamentous or shoot-like gametophytic forms, and the sporophyte comprises a single stem capped in a sporangium [2, 9, 10].The emergence of the vascular plant clade was associated with a shift to sporophyte dominance, a suite of sporophytic innovations including branching, and a gradual reduction in gametophyte size [4, 11–13]. The mechanisms underpinning architectural diversification in each life cycle stage are unknown, but the shared genetic toolkit available to land plants implicates conserved developmental mechanisms [14, 15].

One major candidate for such a conserved mechanism is the regulated intercellular transport of the plant hormone, auxin [16]. Most of our understanding of the key contribution of auxin transport to meristem function and shoot architecture comes from studies in flowering plants [17]. Pharmacological treatments that disrupt auxin transport across the multicellular apical dome inhibit leaf initiation [18], and in *Arabidopsis*, mutations in the auxin efflux carrier *PIN-FORMED1* (*PIN1*) gene cause similar defects [19]. Local application of auxin to naked apices is sufficient to induce leaf initiation, and such auxin maximum formation usually occurs as a result of the dynamic polar transport of auxin by PIN1 to foci on the meristem [18, 20, 21]. Distinct patterns of leaf initiation arise as a consequence of the self-organizing properties of the auxin transport system [22, 23]. Patterns of leaflet initiation [24], vein insertion in leaves [25], marginal ornamentation [26], and leaf growth [27] are similarly regulated by PIN-dependent auxin transport. Thus, PIN-mediated auxin transport acts as a major contributor to architectural diversity in flowering plants by modulating meristem function and leaf development.

Auxin transport assays and auxin transport inhibitor applications in the lycophyte *Selaginella kraussiana* have shown that auxin transport has conserved roles in sporophytic meristem function within the vascular plants [28–31]. Several recent papers have considered the contributions of auxin and its transport to bryophyte development, using mosses as model systems [32–35]. Bulk basipetal polar auxin transport has been demonstrated in moss sporophytes, and application of polar auxin transport inhibitors (PATIs) causes severe disruptions in development, resulting in the formation of multiple sporangia [32, 33]. These data suggest that polar auxin transport is a conserved regulator of sporophyte development, but the extent of conservation between the sporophyte and gametophyte generation is unclear. Although gametophytic auxin transport has been reported in ferns [36], mosses [37, 38], liverworts [39, 40], and charophyte algae [41], it has proved undetectable in the gametophytic shoots of mosses [32, 33]. As sporophytic and gametophytic shoots (gametophores) evolved independently, the convergent shoot morphologies of each generation could have arisen through the recruitment of distinct genetic pathways to regulate development in plant evolution [32, 33].

One hypothesis to account for the divergent auxin transport properties of sporophytic and gametophytic shooting systems in mosses is a divergence in PIN function between mosses and vascular plants or between generations in mosses. In *Arabidopsis*, PIN function depends on subcellular protein localizations; whereas PIN1–PIN4 and PIN7 (canonical PINs) are plasma membrane targeted and function in many developmental processes by regulating intercellular auxin

[6]Co-first author
*Correspondence: cjh97@cam.ac.uk
This is an open access article under the CC BY license (http://creativecommons.org/licenses/by/3.0/).

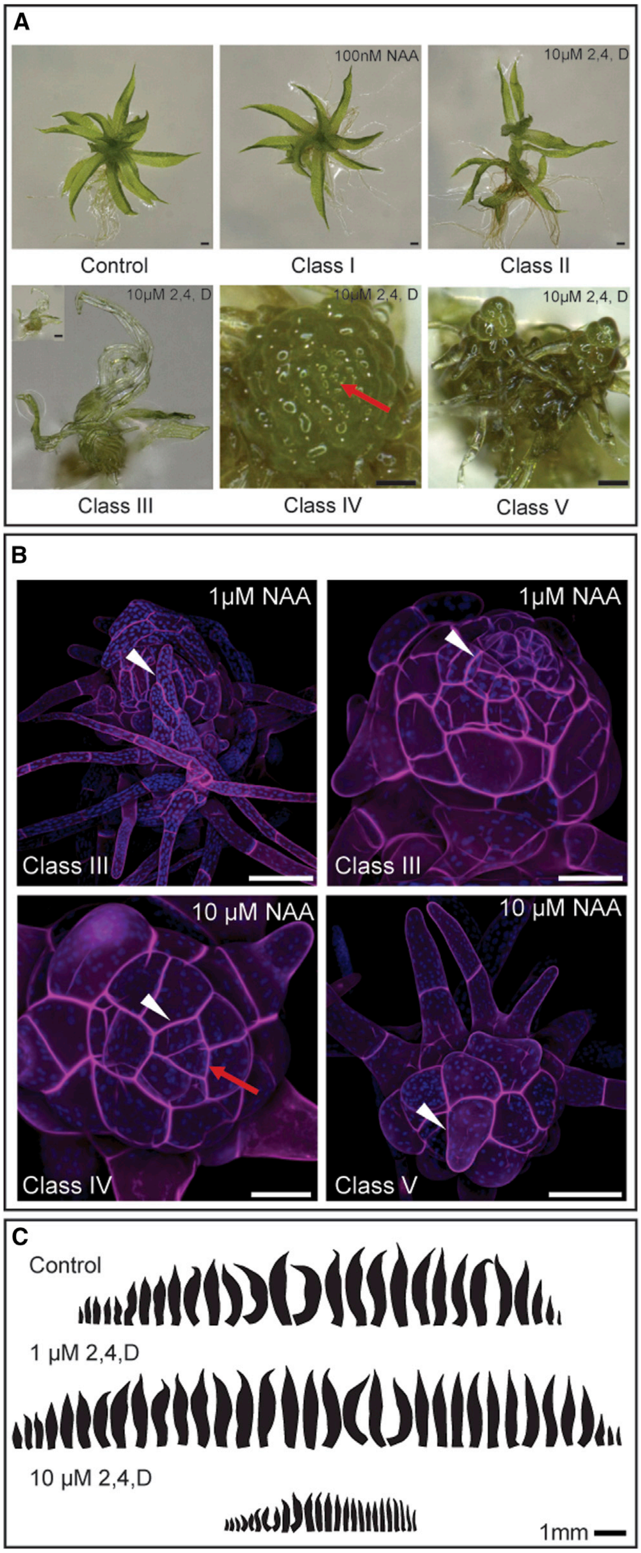

Figure 1. Treatment with Auxins Perturbs Leaf Development and Can Cause Meristem Arrest

Plants were grown on BCD + ammonium tartrate (AT) medium for 3 weeks in continuous light at 23°C.

(A) Developmental defects arising as a result of auxin treatments. Scale bars in untreated control, class I and class II, and inset for class III represent 200 μm; scale bars in class IV and class V represent 100 μm. Red arrow indicates the apical cell.

(B) Confocal micrographs of class III–V buds showing severely stunted leaves (arrowheads in 1 μM NAA treatments), a leaf progenitor cell and apical cell (arrowhead and arrow in class IV shoot), and a rhizoid terminating the shoot (arrowhead in class V shoot). Scale bars represent 50 μm.

(C) Leaf series of plants grown on different auxin treatments. 1 μM 2,4-D mildly promotes leaf initiation and development, whereas 10 μM 2,4-D inhibits leaf initiation and development relative to controls.

transport, PIN5, PIN6, and PIN8 (noncanonical PINs) are ER targeted and are thought to regulate auxin homeostasis within cells [42–44]. The apparent functional divergence between canonical and noncanonical PINs reflects differences in protein structure between the two classes, and canonical PINs have a predicted intracellular domain with characteristic motifs involved in membrane targeting, which is greatly reduced in noncanonical PINs [45, 46]. The genome of the model moss *Physcomitrella patens* encodes four PIN proteins (PINA–PIND), whose localization has been assayed by heterologous expression assays in tobacco protoplasts. These suggested that PINA localizes to the ER and that PIND localizes in the cytosol, implying roles in intracellular auxin homeostasis rather than intercellular transport [34]. Although these data support the hypothesis that the absence of bulk basipetal auxin transport in moss gametophores could reflect a divergence in PIN function between mosses and flowering plants, they cannot account for the divergent auxin transport properties of moss sporophytes and gametophores. Furthermore, we have recently shown that vascular plant PIN proteins diversified from a single canonical ancestor and that three *Physcomitrella* PINs (PINA–PINC) have canonical structure, placing canonical PINs one likely ancestral type within the land plants [45]. The data above raise questions about the evolution of land plant PIN functions and the roles of auxin transport and PIN proteins in moss gametophore development.

Here, we show that *Physcomitrella* PINs are plasma membrane targeted and that PIN-mediated auxin transport regulates many aspects of gametophore development. *pin* mutants have greatly impaired fertility and striking sporophytic defects that are similar to published defects arising from treatment with auxin transport inhibitors. Our results show that PIN proteins are conserved auxin transport facilitators.

Results

Exogenously Applied Auxins Affect Meristem Function and Leaf Development

To clarify the roles of auxin in moss gametophore development, we grew colonies on medium supplemented with auxins that have different biochemical properties: indoleacetic acid (IAA), naphthylacetic acid (NAA), and 2,4-dichlorophenoxyacetic acid (2,4-D). Although weak effects were seen with the native auxin IAA (Figure S1 available online), a spectrum of phenotypes of lesser-to-greater severity was observed in treatments with NAA and 2,4-D and was classified into five phenotypic classes, classes I–V (Figures 1A and S1). An increased frequency of more-severe phenotypes correlated with increasing auxin concentrations (Figure S1C). When grown on lower auxin concentrations (e.g., 100 nM NAA, 1 μM 2,4-D), class I and class II shoots were prevalent. Class I shoots appeared similar to controls, but the zone of rhizoid emergence was displaced apically, as in previous reports [47–49]. Class II shoots (seen in 2,4-D treatments) were elongated and had more leaves than controls (Figures 1A, 1C,

S1A, and S1D). Class III shoots were stunted, producing fewer leaves than untreated controls (Figures 1A, 1B, and S1D), and leaves were narrow with fewer, longer cells than untreated controls (Figures 1C, S1B, and S1D). In class IV shoots, leaf outgrowth was suppressed, and gametophores comprised a raspberry-like dome of cells above a zone of rhizoid emergence (Figure 1A). Confocal microscopy revealed a spiral of successively larger leaf progenitor cells emanating from the apical cell, thus demonstrating its continued activity (Figure 1B). The strongest effect of auxin was revealed in class V shoots, which lost apical cell function. Shoots terminated with irregularly shaped cells, or rhizoids, consistent with previous reports [47, 49] (Figure 1B). These data suggest that accumulation of auxin in shoots triggers diverse developmental effects at different threshold levels. Notably, auxin accumulation causes defects in meristem function, leaf initiation, and oriented leaf growth.

Treatment with Auxin Transport Inhibitors Phenocopies Auxin Treatment

By analogy to flowering plants, we hypothesized that gametophore development is normally driven by changes in the auxin distribution within tissues, which was disrupted by adding exogenous auxin. We reasoned that such changes might occur by a conserved transport-dependent mechanism. To test this hypothesis, we analyzed the effect on gametophore development of the compounds 1-*N*-naphthylphthalamic acid (NPA) and naringenen (Nar), which are potent PATIs in angiosperms. Treatment with NPA caused mild developmental defects in leaves (Figure 2C), and both inhibitors had a similar effect to treatments with 2,4-D, which first promoted and then mildly suppressed leaf development (Figures 2A and 2B; Figure S2 in comparison to Figure 1; Figure S1D). However, class III–V phenotypes were not observed. Although the concentrations of NPA used here strongly inhibit auxin transport in *Arabidopsis*, the effect of PATIs is not well characterized in mosses, and we reasoned that our treatments might only partially inhibit auxin transport. We hypothesized that such partial inhibition might result in relatively mild phenotypes but might sensitize colonies to the addition of exogenous auxin. To test this hypothesis, we treated colonies with 5 μM NPA or Nar together with 100 nM NAA, which by itself only induces class I defects. These treatments gave rise to colonies with few visible gametophores that had class II and III defects (Figures 2A, 2B, S2B, and S2C): further investigation also revealed a number of class IV and V gametophores (Figures 2D and S2B). This response is similar to responses to higher concentrations of auxin applied alone, suggesting that transport normally relieves the effect of applying exogenous auxins.

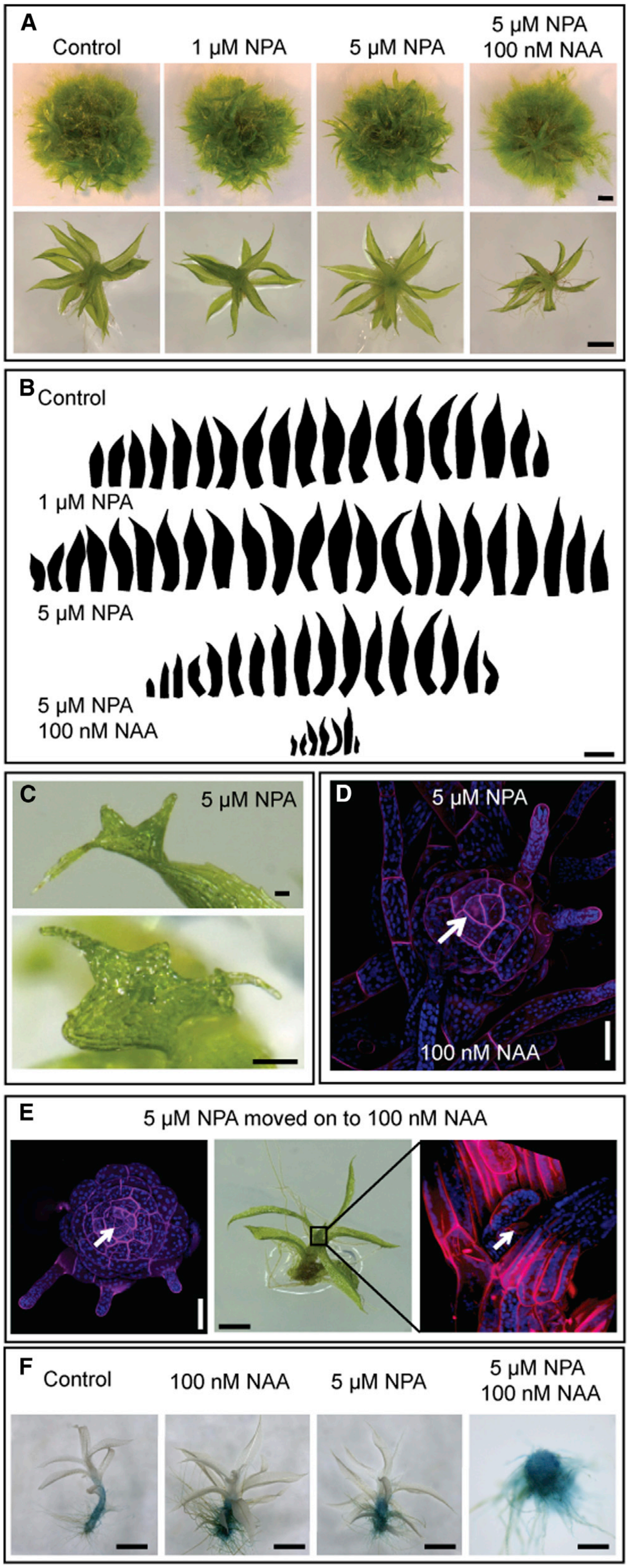

Figure 2. Pharmacological Polar Auxin Transport Inhibition Perturbs Leaf Development and Can Cause Meristem Arrest

Plants were grown on BCD + AT medium supplemented with auxin and transport inhibitors for 3 weeks in continuous light at 23°C.

(A) NPA treatment caused class I or II shoot defects, but used in combination with 100 nM NAA, it caused class III and IV defects. Scale bars represent 1 mm by row.

(B) Leaf series show that 1 μM NPA caused an increase in leaf number and size relative to untreated controls. 5 μM NPA mildly inhibits leaf initiation and development, and addition of 100 nM NAA strengthens the inhibition. The scale bar represents 1 mm.

(C) Treatment with 5 μM NPA caused mild perturbations to leaf development. Scale bars represent 100μm.

(D) Treatment with 5 μM NPA and 100 nM NAA generated class IV shoots. The scale bar represents 50 μM.

(E) If 100 nM NAA was added to plants treated with 5 μM NPA after 2 weeks, shoots that had already initiated arrested, revealing the apical cell (arrow). The scale bar represents 0.5 mm.

(F) Gametophores grown for 3 weeks on control medium and medium supplemented with 100 nM NAA or medium supplemented with 5 μM NPA, or both, were stained for β-glucuronidase activity.

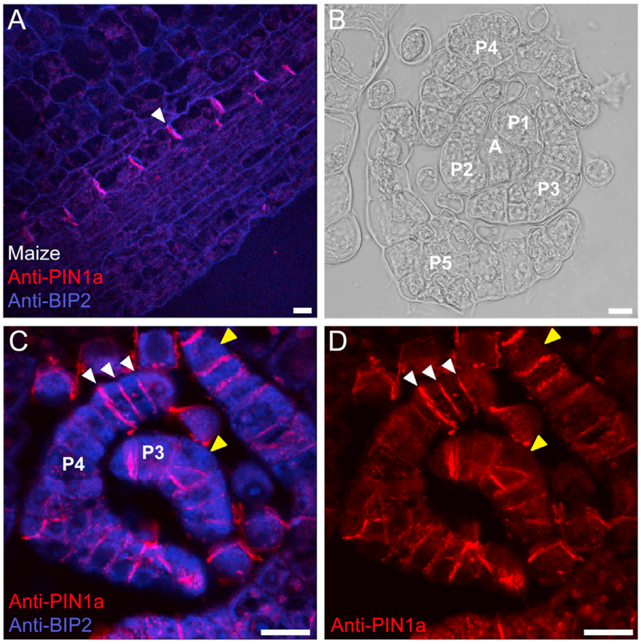

Figure 3. *Physcomitrella* PINs Are Plasma Membrane Targeted

(A) Maize anti-PIN1a antibodies detected a strong polar signal at the plasma membrane in developing maize leaves (arrowhead). The scale bar represents 17.5 μm.

(B) *Physcomitrella* leaves initiate in a spiral around the apical cell, and cell differentiation becomes apparent after P5. The scale bar represents 15 μm.

(C and D) Immunolocalization in *Physcomitrella* leaves showing that anti-BIP2 (blue) and anti-PIN (red) signals do not colocalize and that the PIN signal forms a transverse banding pattern across the youngest leaf primordia around the apex (white arrows). No signal was detected at the outer cell faces (yellow arrows). Scale bars represent 15 μm.

Treatment with Auxin Transport Inhibitors Can Collapse Leaf Development and Meristem Function

The severity of class IV and V responses to auxin made it difficult to determine which aspects of development are disrupted. We therefore varied this treatment by allowing plants to form normal shoots while growing on 5 μM NPA for 2 weeks before adding 100 nM NAA. During the 2 weeks following auxin addition, gametophores underwent progressive developmental arrest. Recently initiated leaves toward the apex became shorter and more slender before initiation ceased, and the apical cell was exposed (Figure 2E). In conjunction with auxin treatments, which promoted or suppressed leaf initiation (Figure S1D), these data suggest that an appropriate auxin level is required for apical cell function and is attained by transport out of the apex.

Treatment with Auxin and Transport Inhibitors Alters the Distribution of a Marker for Auxin Response in *Physcomitrella*

The treatments with auxin and auxin transport inhibitors above suggest that the normal auxin distribution in moss gametophores is transport dependent. To evaluate this hypothesis, we analyzed the staining distribution pattern of an auxin-responsive GH3:GUS reporter [50] in untreated and pharmacologically treated plants (Figure 2F). As in previous reports [32, 50–54], untreated plants accumulated staining at the base of the shoot and in punctuated maxima at points of rhizoid initiation up the shoot. No staining was reproducibly detected in leaves. Treatment with 100 nM NAA increased the density of basal rhizoids and elevated the GUS staining intensity, a response that was phenocopied by treatment with 5 μM NPA. Plants that were grown on 5 μM NPA and 100 nM NAA and had class IV shoot defects accumulated stain at the shoot apex, supporting the inference that auxin transport maintains auxin levels at the apex to regulate its activity.

Physcomitrella PINs Are Plasma Membrane Localized

On the basis of the data above, we reasoned that the auxin distribution in gametophore apices and leaves might be PIN regulated. We therefore used an immunolocalization approach with transverse sections just above the apex to determine where *Physcomitrella* PINs localize (Figure 3B). We used antibodies raised in guinea pigs against residues 264–413 or 264–411 of maize PIN1-like variants PIN1a and PIN1b, respectively, and, as expected on the basis of published work [55], found that both antibodies gave strong polar plasma membrane-targeted signal in maize leaf sections used as a positive control (Figures 3A and S3). We used an antibody against an abundant ER-targeted protein, BIP2, as a control to test for ER colocalization. In our moss experiments, we found that the BIP2 signal (blue) localized broadly across the undifferentiated leaf tissues of P1–P5 (Figure 3C). In contrast, the PIN signal (red) was restricted mainly to narrow bands spanning the adaxial-abaxial leaf axis at the junctions between cells and did not colocalize with the BIP2 signal (Figures 3C and 3D). We also detected signal on the internal faces of cells around the presumptive midvein, but signal at the outermost cell edges was absent. Thus, *Physcomitrella* PINs are plasma membrane targeted, can polarize, and localize in tissues that are responsive to disruption of auxin levels.

Physcomitrella pin Mutants Phenocopy Plants Treated with Auxin or Auxin Transport Inhibitors

Physcomitrella PINs A–C are canonical and share many sequence motifs with *Arabidopsis* PIN1 in the central intracellular loop, whereas PIND is highly divergent [45], and *PINA* and *PINB*, but not *PINC*, were strongly expressed in gametophores (Figures S4A and S4B). Therefore, to analyze PIN function in *Physcomitrella*, we engineered targeted disruptants for *PINA* and *PINB* by homologous recombination [56] (Figures S4C–S4E). Several lines with the same phenotypes were recovered for each insertion, suggesting that mutant phenotypes were caused by lesions in targeted loci (Figure S4F). RT-PCR showed that disrupted *PINA* and *PINB* transcripts were present at low levels in *pinA*, *pinB*, and *pinA pinB* double mutants (Figures S4G and S4H), suggesting that the mutants may not be null. *pinA* and *pinB* single mutant shoots were not obviously different from wild-type (WT) (Figures 4A and 4B), but quantitative analysis showed that *pinB* gametophores were longer than WT (Figure S5). Double disruptants had class II shoot defects and defects in oriented leaf growth and cell division (Figures 4A and S5). *pinA pinB* double mutants therefore resemble plants treated with auxin (Figure S1), suggesting that they accumulate auxin as a result of a deficiency in auxin transport.

Physcomitrella pin Mutants Are Hypersensitive to Auxin

The *pinA pinB* double mutant phenotype comprises class II defects, but more-severe defects were not observed. We reasoned that this may be due to residual PINC activity or residual activity in other components of the auxin transport pathway, such as PGP or ABC transporters [57]. We also

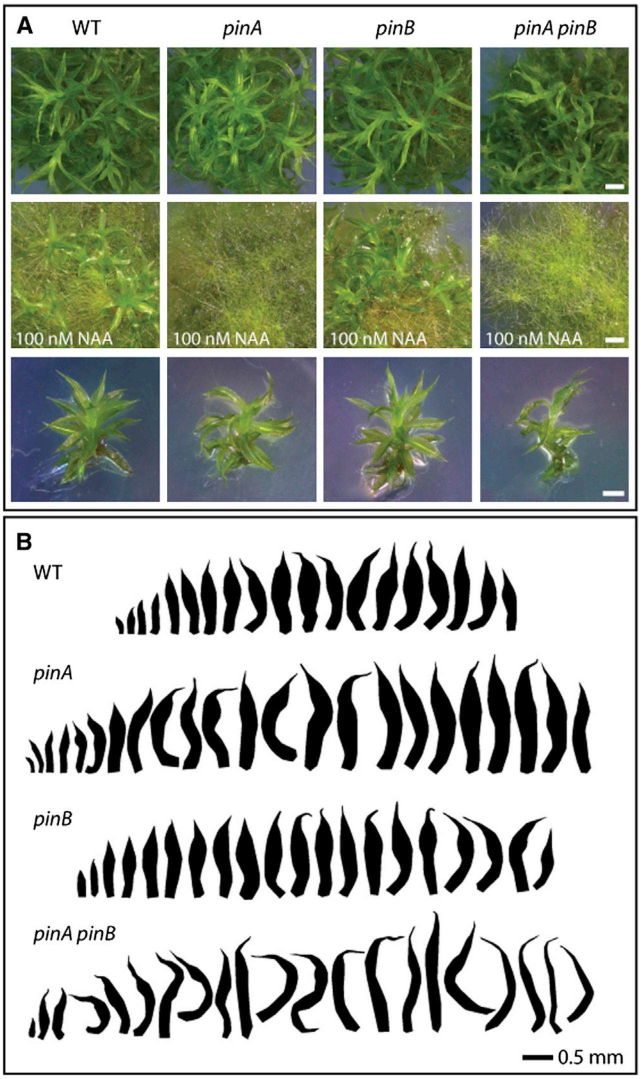

Figure 4. *Physcomitrella* PIN Proteins Regulate Leaf Initiation and Development

Plants were grown on BCD + AT medium for 3 weeks in continuous light at 23°C.

(A) Whereas *pinA* and *pinB* mutants are not easily distinguished from WT, *pinA pinB* mutants have class II shoot defects. *pinA* mutants and *pinA pinB* mutants are sensitized to NAA. The scale bar represents 1 mm by row.

(B) Leaf series show subtle differences in leaf shape and size between WT and single mutants, whereas *pinA pinB* mutants have conspicuously irregularly shaped leaves that are longer and thinner than WT.

reasoned that if we had reduced the auxin transport capacity, mutants would be more sensitive to exogenous auxin treatment than WT plants. To test this hypothesis, we grew mutants on 100 nM NAA for 4 weeks. In *pinA* and *pinA pinB* mutants, this treatment generated gametophores with class III–V phenotypes (Figure 4A), phenocopying the effect of NAA and NPA cotreatment (Figure 2A). Our results suggest that PINA and PINB act redundantly to remove auxin from the apex and initiating leaves, allowing normal development to proceed. As shoot development is strongly affected in *pinA* single mutants treated with 100 nM NAA, but not in *pinB* mutants, we postulate that PINA plays the dominant role (Figure S4B). These data support the hypothesis that the apical auxin distribution in *Physcomitrella* regulates gametophore architecture and is modulated by PIN proteins.

A Marker for Auxin Response Is Redistributed in *Physcomitrella pin* Mutants

To further test the hypothesis that PIN proteins modulate the auxin distribution in *Physcomitrella*, we analyzed the staining distribution pattern of the GH3:GUS reporter [50] in WT and mutant plants (Figure 5A). In *pinA* and *pinB* single mutant shoots, staining was slightly stronger than in WT and displaced up the stem. In contrast, the staining intensity in *pinA pinB* mutants was strongly reduced with respect to WT and single mutants and, where present, was localized to the middle portion of the stem. Gametophores with the most-severe leaf phenotypes had the least signal and very few rhizoids initiated; no basal zone of rhizoid emergence was apparent (Figures 5A–5C). Transverse sections taken through the base and midstem region confirmed this inference, indicating a difference in the apical-basal auxin level and distribution as the main defect (Figures 5B and 5C). To test whether auxin-inducible phenotypic alterations to shoot development (Figure 3A) corresponded to an altered auxin response distribution, plants were grown on 100 nM NAA before staining. Whereas gametophores with a class I–III response showed only an upregulation in signal intensity, *pinA* and *pinA pinB* mutants with class IV and V phenotypes accumulated staining toward or at the apex (Figure 5A). These data support the hypothesis that PIN proteins modulate the auxin distribution in gametophores.

Physcomitrella pin Mutants Have Disrupted Tropic Responses

In angiosperms, PIN-mediated polar auxin transport drives phototropic and gravitropic responses in shoots and roots [58, 59]. *Physcomitrella* filaments and gametophores have strong negative gravitropism when grown in the dark [60]. Interestingly, moss mutants defective in filament gravitropism are not defective in shoot gravitropism, suggesting that two distinct tropism pathways may operate [60]. To assess a putative role for PIN-mediated auxin transport in gravitropism, we grew WT, single and double *pin* mutants for 2 weeks in the light and then grew them vertically in the dark on sucrose supplemented medium (0.5% w/v) for a further 2 weeks. In WT plants, this treatment induced a strong negative gravitropic response in both filaments and gametophores (Figures 6A–6C). Whereas *pinA* and *pinB* single mutants showed a normal gravitropic response, the *pinA pinB* double mutant had agravitropic gametophores. This result was phenocopied by treatment with 2,4-D (data not shown). To assess a putative role in phototropism, we grew plants as above but then exposed them to a unidirectional blue light stimulus for 24 hr. Whereas the tips of WT gametophores showed a clear reorientation toward the light stimulus (Figure 6D), *pinA pinB* colonies subjected to the same light stimulus continued to grow in a disoriented manner, showing no clear tropic growth toward the light stimulus (Figure 6D). These data suggest conservation of PIN-dependent, auxin transport-driven gravitropism and phototropism pathways between mosses and angiosperms and again highlight the importance of auxin transport-driven processes in *Physcomitrella* gametophore development.

Physcomitrella pin Mutants Have Disrupted Sporophyte Development

For reasons outlined in the introduction, this study has principally targeted recent controversy surrounding the roles of

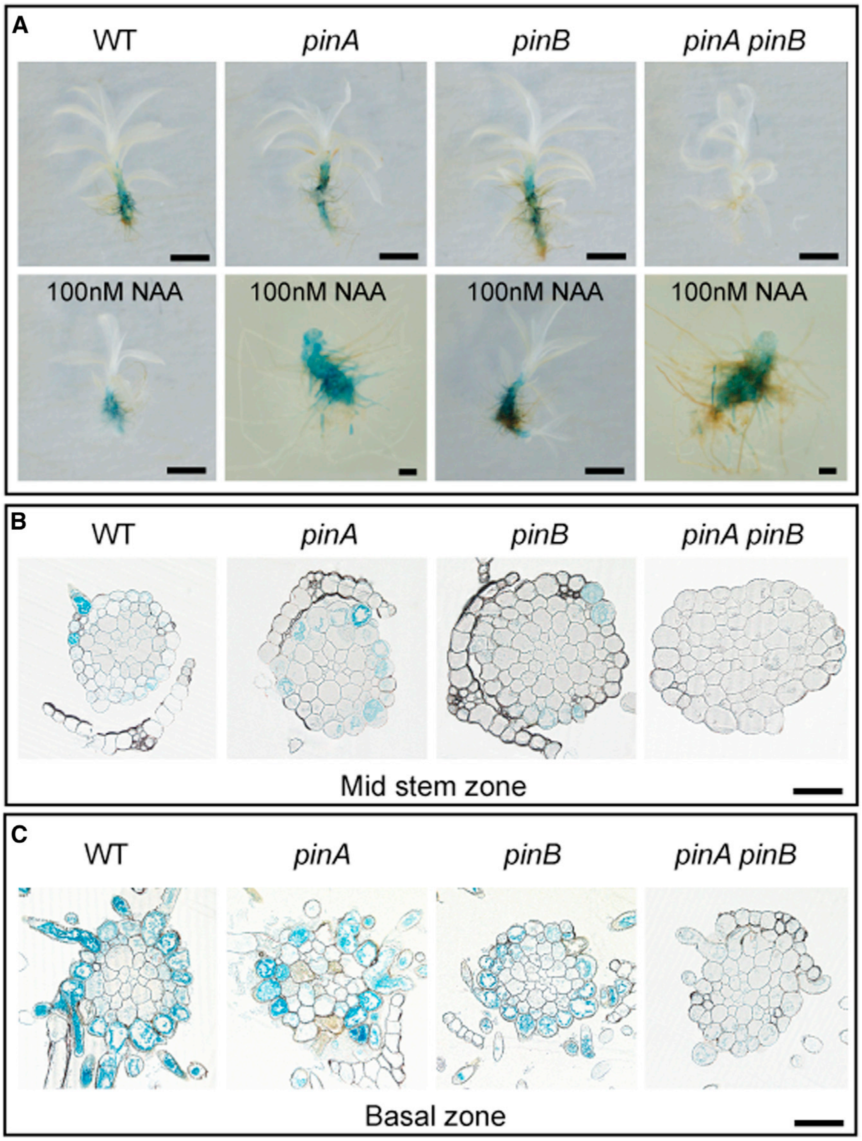

Figure 5. A GH3:GUS Reporter Is Redistributed in *pinA pinB* Mutant Shoots

(A) GH3:GUS expression in the WT, *pinA*, *pinB*, and *pinA pinB* lines was assessed after 3 weeks of growth on control medium (top row) or medium supplemented with 100 nM NAA (bottom row). Gametophores were extracted and then stained for β-glucuronidase activity for 30 min. Scale bars (long) represent 1 mm; scale bars (short) represent 100 μm.

(B) Transverse sections through the midstem region showed a patterned distribution of epidermal staining in WT, *pinA* and *pinB* plants. In *pinA pinB* mutants, the staining intensity was much reduced. The scale bar (long) represents 1 mm

(C) Transverse sections through the basal region showed strong, evenly distributed epidermal staining in WT, *pinA* and *pinB* plants. In *pinA pinB* mutants, the staining intensity was much reduced or absent. The scale bar (long) represents 1 mm

auxin transport in *Physcomitrella* gametophore development. However, as auxin transport has previously been detected in moss sporophytes and application of transport inhibitors perturbs sporophyte development [32], we also tested the hypothesis that PIN-mediated auxin transport regulates sporophyte development. We detected sporophytic expression of *PINA* and *PINB* (Figure S4B) and grew WT and *pin* mutant sporophytes to evaluate their phenotypes. Cultures were grown on four peat plugs in continuous light at 23°C for 6 weeks before transfer to a short-day 16°C regime for induction, and all the sporophytes present were harvested 4 weeks after induction. Whereas gametangia appeared normal (Figure 7A), PINA and PINB contributed synergistcially to fertility and development (Figures 7B and S6). Sporophytic defects were detected with variable penetrance: a low proportion (6 out of 208) on our GH3:GUS WT line had duplicated sporangia or dead sporophytes. Whereas *pinA* mutants had no obvious defects (1 out of 115 had duplicated sporangia; 3 out of 115 had an enlarged sporangium), a significant proportion of *pinB* mutants had duplicated sporangia (19 out of 89; 6 out of 89 were dead or had other defects), and around half of *pinA pinB* mutants had severe, sometimes lethal, developmental defects (5 out of 34 had duplicated sporangia; 7 out of 34 were dead or had other defects). The results suggest that PIN-mediated auxin transport regulates sporophytic shoot development, with a stronger contribution from *PINB* than from *PINA*.

Discussion

Physcomitrella PINs Can Polarize at the Plasma Membrane

On the basis of heterologous gene expression assays in tobacco, previous work suggested that *Physcomitrella* PINs A and D localize at the ER and cytosol, respectively, and land plant PINs were therefore postulated to have an ancestral role in regulating intracellular auxin homeostasis rather than intercellular transport [34, 35]. However, we have recently shown that *Physcomitrella* PINA–PINC are canonical, sharing sequence motifs that are required for plasma membrane targeting with *Arabidopsis* canonical PINs [45]. Our work suggested that canonical PINs are one ancestral type within the land plants and that *Physcomitrella* PINs A–C should have a capacity for plasma membrane targeting [45]. Using immunolocalization, we have found that *Physcomitrella* PINs A–C can indeed target the plasma membrane; we did not detect signal elsewhere in cells, and we did not detect signal colocalizing with an ER marker.

Physcomitrella PIN localization usually formed a conspicuous banding pattern traversing the adaxial-abaxial leaf axis, where two cells contact one another (Figures 3 and S3). Where leaves were thickened around the midvein, we also detected signal on the cell faces that were in contact with other cells, but the outermost cell faces were usually free from signal. Although we cannot rule out the possibility that each neighboring cell contributes to the high signal intensity at cell junctions, in our view, the localization is polarized. As auxin-treated

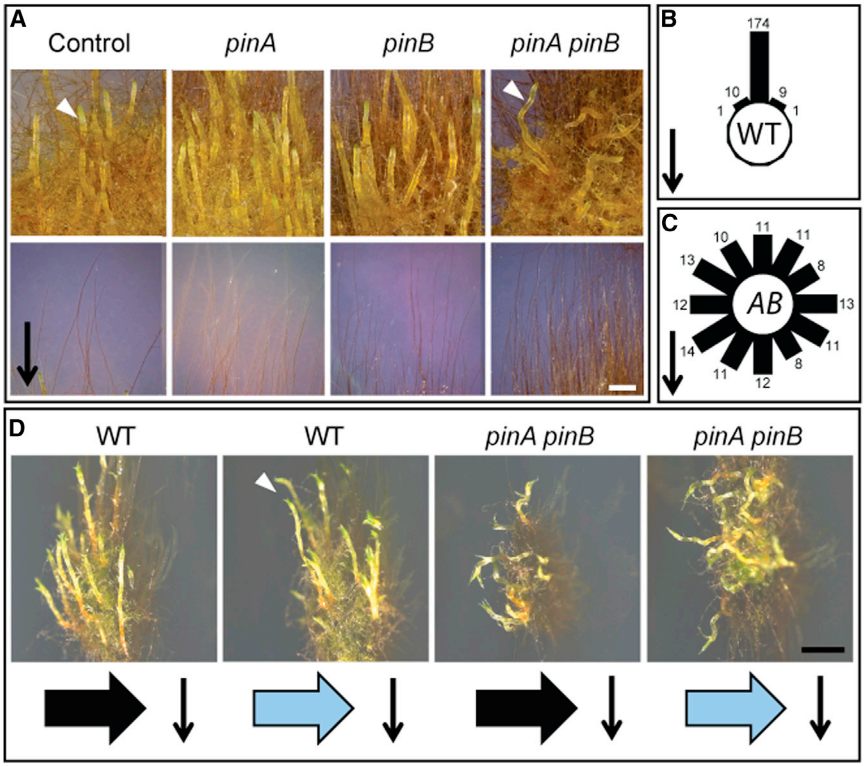

Figure 6. PIN Proteins Mediate *Physcomitrella* Shoot Tropism

Plants were grown on BCD + AT medium for 3 weeks horizontally in continuous light at 23°C before plates were wrapped in foil, oriented vertically, and allowed to grow for 2 more weeks.

(A) Whereas filaments reoriented away from the new gravity vector in all genotypes, shoot gravitropism was abolished in *pinA pinB* mutants. The scale bar represents 500 μm.

(B and C) For WT (B) and *pinA pinB* (C), the response was quantified by counting the number of shoot tips in 30° sectors relative to the gravity vector.

(D) Dark-grown colonies of WT and *pinA pinB* mutant plants were exposed to unidirectional blue light (blue arrows) for 24 hr to assess the phototropic response of etiolated gametophores. Whereas control shoots were kept in the dark and maintained their previous growth vectors (vertical arrows), WT shoots reoriented toward the light source (arrowhead). *pinA pinB* mutant shoots showed no obvious reorientation. The scale bar represents 500 μm.

gametophores and *pinA pinB* mutants have around half the number of cells in the mediolateral leaf axis than normal and the mediolateral leaf axis is elaborated by asymmetric cell divisions [61], a polar localization pattern perpendicular to the mediolateral axis is consistent with a role for PINA and PINB in promoting asymmetric cell division. These results suggest a role for canonical *Physcomitrella* PINs in intercellular polar auxin transport in leaf development.

PIN-Mediated Auxin Transport Drives Meristem Function and Leaf Development

Recent work was unable to detect polar auxin transport in gametophytic moss shoots, and no effect of treatment with transport inhibitors was observed, leading to the conclusion that auxin transport does not contribute to gametophore development [32, 33]. We were also unable to detect long-range polar auxin transport using radio-labeled IAA (data not shown). The discrepancy between the results that we obtained with NPA and previously published results arises from a difference in experimental approach. Whereas previous experiments immersed fully grown shoots in 50 μM NPA [32, 33], we grew colonies on NPA, exposing shoots to transport inhibition from the earliest developmental stages, and cotreatment with low auxin concentrations was needed to see strong developmental effects (Figure 2). We found that treatment of WT gametophores with NPA disrupted extension of proximodistal and mediolateral axes of leaf development and disrupted meristem function. The effects observed were similar to treatments with high concentrations of auxin or treatments of *pinA* mutants with low concentrations of auxin. Again, these results support a role for PIN-mediated auxin transport in the asymmetric cell divisions that drive leaf development and meristem function [61]. Consistent with PIN localization patterns, we hypothesize that auxin transport in moss gametophores occurs in a localized manner, to remove auxin from the leaves and meristem without detectable long-distance flux [62]. It is also possible that *Physcomitrella* PINs distribute auxin principally in the epidermis and, therefore, that the overall levels of transport involved are low.

PIN-Regulated Shoot Development Is a Deep Homology of Stomatophytes

Collectively, our data show that auxin transport regulates a suite of characteristics in *Physcomitrella* gametophore development that are similar to the developmental characteristics that are PIN regulated in angiosperm sporophytes, and our inferences are supported by data from Viaene et al. [63], published in this issue of *Current Biology*. PIN-mediated auxin transport in *Physcomitrella* regulates intrinsic developmental processes, such as asymmetric cell division, growth, meristem function, and leaf development, and dynamic responses to the environment, such as shoot tropisms. In conjunction with recently published results showing that charophytes have a capacity for long-range polar auxin transport [41], the regulation of these aspects of gametophore development in *Physcomitrella* raises the possibility that auxin transport could be a core mechanism for plant development that was recruited from the gametophyte to the sporophyte during land plant evolution. Alternatively, the roles of PIN-mediated auxin transport could have evolved convergently in moss gametophores. In either case, the recruitment of PIN-mediated auxin transport to regulate gametophore development is a clear instance of deep homology within the stomatophytes and the first that affects such general developmental programs.

PIN-Mediated Auxin Transport Is a Conserved Regulator of Sporophyte Development

Work in *Selaginella* has shown that the roles of polar auxin transport in regulating apical meristem function and shoot branching are conserved within the vascular plants [28–31]. Previous work in mosses has shown that bulk polar auxin

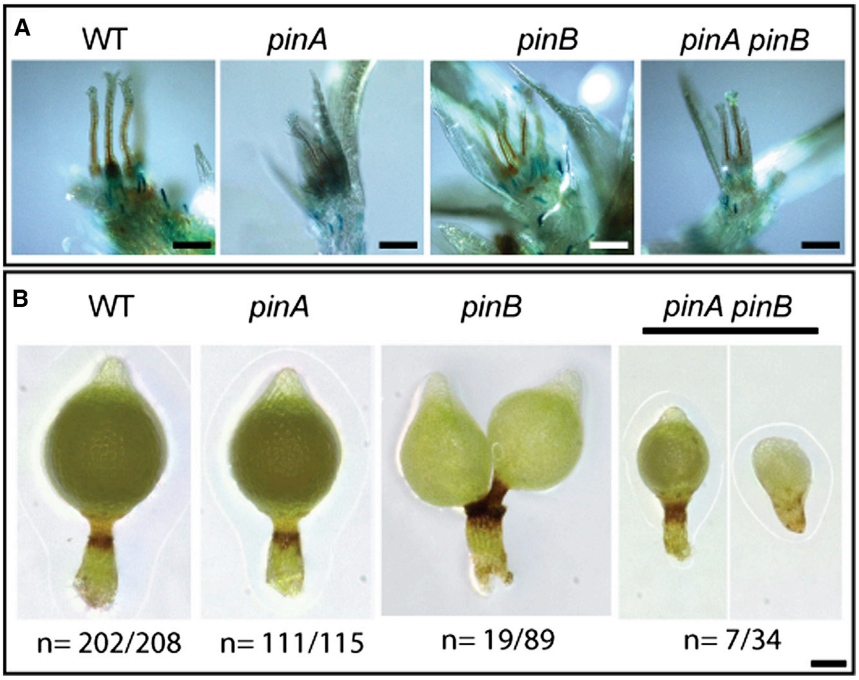

Figure 7. PIN Proteins Regulate *Physcomitrella* Sporophyte Development

Plants were grown on peat plugs in continuous light for 6 weeks at 23°C before transfer to a short-day regime at 16°C. All visible sporophytes were dissected out of gametophores after a further 4 weeks and photographed using a Keyence VHX-1000 microscope.

(A) Gametangium development appeared normal. Scale bars represent 75 μM.

(B) Gross phenotypic perturbations were rare in WT or *pinA* lines but occurred with variable penetrance in *pinB* and *pinA pinB* lines. The scale bar represents 100 μM.

transport in sporophytes can be disrupted by NPA treatment, causing multiple sporangia to form [32, 33]. Our data also support the notion that sporophyte development in *Physcomitrella* is regulated by polar auxin transport [32, 33]. We have demonstrated that *PINA* and *PINB* are expressed in sporophytes and contribute synergistically to fertility and development (Figure 7); PIN-mediated auxin transport is a conserved regulator of sporophyte development in stomatophytes. We note that the duplicated sporangium phenotype of *pinB* and *pinA pinB* mutants reproduces branching morphologies of early prevascular fossils, such as *Partitatheca* [13], and speculate that this phenotype could arise by an early embryonic duplication of the apical cell, or bifurcation [64–66]. PIN-mediated auxin transport is a major driver of plant architecture in flowering plants [17], and changes in meristem function underpin architectural divergence between plant groups [4, 67]. The identification of conserved roles for auxin transport in land plant meristem function opens the possibility that PIN proteins played a key role in the radiation of plant form.

Experimental Procedures

A GH3:GUS reporter line [50] was used as the WT moss strain. Spot cultures were grown as described previously [61], and tissue for genetic analysis was prepared as in [50]. All lines were stored in the International Moss Stock Center (http://www.moss-stock-center.org; see Supplemental Information).

For immunolocalizations, tissue was grown for 4 weeks in continuous light, fixed in 3:1 methanol acetic acid, dehydrated, and embedded in PEG 1600. Eight-micrometer sections were interrogated with anti-maize PIN antibodies [55] at a 1/150 dilution and anti-BIP2 (Agrisera) at a 1/50 dilution. DyLight 594 and DyLight 405 were used as secondary antibodies at a 1/300 dilution.

pin disruptants were generated and screened for insertion as described in Supplemental Information.

GUS staining was carried out as elsewhere [32]. Light micrographs were compiled using a Keyence VHX-1000 series microscope with 50× and 200× objectives. Confocal imaging was undertaken as previously described [61], except for immunolocalizations; a Leica TCS 5 was used, with excitation from the Diode 405 and HeNe 594 laser lines, and emission was collected at 410–480 nm and 600–670 nm.

Supplemental Information

Supplemental Information includes Supplemental Experimental Procedures and six figures and can be found with this article online at http://dx.doi.org/10.1016/j.cub.2014.09.054.

Author Contributions

E.L.D., R.R., and C.J.H. conceived this study. All authors contributed to experimental design. Foundational experiments were undertaken by T.A.B., M.M.L., T.A., N.M.B., M.B., X.Y.W., C.D.W., and C.J.H., with supervision from E.L.D., R.R., and C.J.H. T.A.B. contributed Figures 6B–6D, S1C, and S2B; M.M.L. contributed Figures 5B and 5C; Y.C. contributed Figure 7B; T.A. contributed Figures S4G and S4H; R.J.D. contributed Figures S1D, S2A, and S5; E.L.D. contributed Figure S4A; C.D.W. contributed Figure S4B; X.Y.W. contributed Figure S4F; and C.J.H. contributed the remainder. T.A.B., M.M.L., T.A., R.J.D., E.L.D., R.R., and C.J.H. contributed to data analysis and interpretation. The final manuscript was drafted by C.J.H., with help from T.A.B., T.A., E.L.D., and R.R. C.J.H. handled submission. D.O. contributed anti-PIN antibodies and technical help with immunohistochemistry.

Acknowledgments

We thank James Lloyd for a preliminary experiment. We thank Gertrud Wiedemann and Anna Beike for initial expression analyses and Ingrid Heger and Agnes Novakovic for technical assistance. We thank Jane Langdale and David Baulcombe for comments on the manuscript. C.J.H. is supported by a Royal Society University Research Fellowship, a Gatsby Charitable Foundation Fellowship (GAT2962), and the Biotechnology and Biological Sciences Research Council (BB/L00224811), and R.R. is supported by the Deutsche Forschungsgemeinschaft (SPP 1067, RE 837/6) and the Excellence Initiative of the German Federal and State Governments (EXC294).

Received: December 18, 2012
Revised: September 3, 2014
Accepted: September 22, 2014
Published: November 13, 2014

References

1. Harrison, C.J., Alvey, E., and Henderson, I.R. (2010). Meiosis in flowering plants and other green organisms. J. Exp. Bot. *61*, 2863–2875.

2. Graham, L.E., Cook, M.E., and Busse, J.S. (2000). The origin of plants: body plan changes contributing to a major evolutionary radiation. Proc. Natl. Acad. Sci. USA *97*, 4535–4540.
3. Gensel, P.G. (2008). The earliest land plants. Annu. Rev. Ecol. Evol. Syst. *39*, 459–477.
4. Langdale, J.A., and Harrison, C.J. (2008). Developmental changes during the evolution of plant form. In Evolving Pathways: Key Themes in Evolutionary Developmental Biology, A.M.G. Fusco, ed. (Cambridge: Cambridge University Press), pp. 299–315.
5. Friedman, W.E. (2013). Plant science. One genome, two ontogenies. Science *339*, 1045–1046.
6. Qiu, Y.-L., Li, L., Wang, B., Chen, Z., Knoop, V., Groth-Malonek, M., Dombrovska, O., Lee, J., Kent, L., Rest, J., et al. (2006). The deepest divergences in land plants inferred from phylogenomic evidence. Proc. Natl. Acad. Sci. USA *103*, 15511–15516.
7. Karol, K.G., McCourt, R.M., Cimino, M.T., and Delwiche, C.F. (2001). The closest living relatives of land plants. Science *294*, 2351–2353.
8. Chang, Y., and Graham, S.W. (2011). Inferring the higher-order phylogeny of mosses (Bryophyta) and relatives using a large, multigene plastid data set. Am. J. Bot. *98*, 839–849.
9. Parihar, N.S. (1967). Bryophyta (Allahabad: Indian Universities Press).
10. Kato, M., and Akiyama, H. (2005). Interpolation hypothesis for origin of the vegetative sporophyte of land plants. Taxon *54*, 443–450.
11. Doyle, J.A. (2013). Phylogenetic analyses and morphological innovations in land plants. In The Evolution of Plant Form, B.A. Ambrose and M. Purugganan, eds. (Oxford: Wiley-Blackwell), pp. 1–50.
12. Goffinet, B., and Buck, W.R. (2013). The evolution of body form in bryophytes. In The Evolution of Plant Form, B.A. Ambrose and M. Purugganan, eds. (Oxford: Wiley-Blackwell), pp. 51–90.
13. Edwards, D., Morris, J.L., Richardson, J.B., and Kenrick, P. (2014). Cryptospores and cryptophytes reveal hidden diversity in early land floras. New Phytol. *202*, 50–78.
14. Floyd, S.K., and Bowman, J.L. (2007). The ancestral developmental toolkit of land plants. Int. J. Plant Sci. *168*, 1–35.
15. Rensing, S.A., Lang, D., Zimmer, A.D., Terry, A., Salamov, A., Shapiro, H., Nishiyama, T., Perroud, P.F., Lindquist, E.A., Kamisugi, Y., et al. (2008). The *Physcomitrella* genome reveals evolutionary insights into the conquest of land by plants. Science *319*, 64–69.
16. Cooke, T.J., Poli, D., and Cohen, J.D. (2003). Did auxin play a crucial role in the evolution of novel body plans during the late silurian-early devonian radiation of land plants. In The Evolution of Plant Physiology, A.R. Hemsley and I. Poole, eds. (London: Academic Press), pp. 85–107.
17. Reinhardt, D., and Kuhlemeier, C. (2002). Plant architecture. EMBO Rep. *3*, 846–851.
18. Reinhardt, D., Mandel, T., and Kuhlemeier, C. (2000). Auxin regulates the initiation and radial position of plant lateral organs. Plant Cell *12*, 507–518.
19. Gälweiler, L., Guan, C., Müller, A., Wisman, E., Mendgen, K., Yephremov, A., and Palme, K. (1998). Regulation of polar auxin transport by AtPIN1 in *Arabidopsis* vascular tissue. Science *282*, 2226–2230.
20. Reinhardt, D., Pesce, E.-R., Stieger, P., Mandel, T., Baltensperger, K., Bennett, M., Traas, J., Friml, J., and Kuhlemeier, C. (2003). Regulation of phyllotaxis by polar auxin transport. Nature *426*, 255–260.
21. Heisler, M.G., Ohno, C., Das, P., Sieber, P., Reddy, G.V., Long, J.A., and Meyerowitz, E.M. (2005). Patterns of auxin transport and gene expression during primordium development revealed by live imaging of the *Arabidopsis* inflorescence meristem. Curr. Biol. *15*, 1899–1911.
22. Jönsson, H., Heisler, M.G., Shapiro, B.E., Meyerowitz, E.M., and Mjolsness, E. (2006). An auxin-driven polarized transport model for phyllotaxis. Proc. Natl. Acad. Sci. USA *103*, 1633–1638.
23. Smith, R.S., Guyomarc'h, S., Mandel, T., Reinhardt, D., Kuhlemeier, C., and Prusinkiewicz, P. (2006). A plausible model of phyllotaxis. Proc. Natl. Acad. Sci. USA *103*, 1301–1306.
24. Barkoulas, M., Hay, A., Kougioumoutzi, E., and Tsiantis, M. (2008). A developmental framework for dissected leaf formation in the *Arabidopsis* relative *Cardamine hirsuta*. Nat. Genet. *40*, 1136–1141.
25. Scarpella, E., Barkoulas, M., and Tsiantis, M. (2010). Control of leaf and vein development by auxin. Cold Spring Harb. Perspect. Biol. *2*, a001511.
26. Bilsborough, G.D., Runions, A., Barkoulas, M., Jenkins, H.W., Hasson, A., Galinha, C., Laufs, P., Hay, A., Prusinkiewicz, P., and Tsiantis, M. (2011). Model for the regulation of *Arabidopsis thaliana* leaf margin development. Proc. Natl. Acad. Sci. USA *108*, 3424–3429.
27. Kuchen, E.E., Fox, S., de Reuille, P.B., Kennaway, R., Bensmihen, S., Avondo, J., Calder, G.M., Southam, P., Robinson, S., Bangham, A., and Coen, E. (2012). Generation of leaf shape through early patterns of growth and tissue polarity. Science *335*, 1092–1096.
28. Williams, S. (1937). Correlation phenomena and hormones in *Selaginella*. Nature *139*, 966.
29. Wochok, Z.S., and Sussex, I.M. (1973). Morphogenesis in *selaginella*: auxin transport in the stem. Plant Physiol. *51*, 646–650.
30. Wochok, Z.S., and Sussex, I.M. (1975). Morphogenesis in *Selaginella*. III. Meristem determination and cell differentiation. Dev. Biol. *47*, 376–383.
31. Sanders, H.L., and Langdale, J.A. (2013). Conserved transport mechanisms but distinct auxin responses govern shoot patterning in *Selaginella kraussiana*. New Phytol. *198*, 419–428.
32. Fujita, T., Sakaguchi, H., Hiwatashi, Y., Wagstaff, S.J., Ito, M., Deguchi, H., Sato, T., and Hasebe, M. (2008). Convergent evolution of shoots in land plants: lack of auxin polar transport in moss shoots. Evol. Dev. *10*, 176–186.
33. Fujita, T., and Hasebe, M. (2009). Convergences and divergences in polar auxin transport and shoot development in land plant evolution. Plant Signal. Behav. *4*, 313–315.
34. Mravec, J., Skůpa, P., Bailly, A., Hoyerová, K., Krecek, P., Bielach, A., Petrásek, J., Zhang, J., Gaykova, V., Stierhof, Y.D., et al. (2009). Subcellular homeostasis of phytohormone auxin is mediated by the ER-localized PIN5 transporter. Nature *459*, 1136–1140.
35. Viaene, T., Delwiche, C.F., Rensing, S.A., and Friml, J. (2013). Origin and evolution of PIN auxin transporters in the green lineage. Trends Plant Sci. *18*, 5–10.
36. Albaum, H.G. (1938). Inhibitions due to growth hormones in fern prothallia and sporophytes. Am. J. Bot. *25*, 124–133.
37. Reski, R. (1998). Development, genetics and molecular biology of mosses. Bot. Acta *111*, 1–15.
38. Decker, E.L., Frank, W., Sarnighausen, E., and Reski, R. (2006). Moss systems biology en route: phytohormones in *Physcomitrella* development. Plant Biol (Stuttg) *8*, 397–405.
39. Gaal, D.J., Dufresne, S.J., and Maravolo, N.C. (1982). Transport of 14C-indoleacetic acid in the hepatic *Marchantia polymorpha*. Bryologist *85*, 410–418.
40. Larue, C.D., and Narayanaswami, S. (1957). Auxin inhibition in the liverwort *Lunularia*. New Phytol. *56*, 61–70.
41. Boot, K.J., Libbenga, K.R., Hille, S.C., Offringa, R., and van Duijn, B. (2012). Polar auxin transport: an early invention. J. Exp. Bot. *63*, 4213–4218.
42. Benjamins, R., and Scheres, B. (2008). Auxin: the looping star in plant development. Annu. Rev. Plant Biol. *59*, 443–465.
43. Paponov, I.A., Teale, W.D., Trebar, M., Blilou, I., and Palme, K. (2005). The PIN auxin efflux facilitators: evolutionary and functional perspectives. Trends Plant Sci. *10*, 170–177.
44. Krecek, P., Skupa, P., Libus, J., Naramoto, S., Tejos, R., Friml, J., and Zazímalová, E. (2009). The PIN-FORMED (PIN) protein family of auxin transporters. Genome Biol. *10*, 249.
45. Bennett, T., Brockington, S.F., Rothfels, C., Graham, S.W., Stevenson, D., Kutchan, T., Rolf, M., Thomas, P., Wong, G.K.-S., Leyser, O., et al. (2014). Paralogous radiations of PIN proteins with multiple origins of noncanonical PIN structure. Mol. Biol. Evol. *31*, 2042–2060. http://dx.doi.org/10.1093/molbev/msu147.
46. Ganguly, A., Park, M., Kesawat, M.S., and Cho, H.-T. (2014). Functional analysis of the hydrophilic loop in intracellular trafficking of *Arabidopsis* PIN-FORMED Proteins. Plant Cell *26*, 1570–1585.
47. Ashton, N.W., Grimsley, N.H., and Cove, D.J. (1979). Analysis of gametophytic development in the moss, *Physcomitrella patens*, using auxin and cytokinin resistant mutants. Planta *144*, 427–435.
48. Menand, B., Yi, K., Jouannic, S., Hoffmann, L., Ryan, E., Linstead, P., Schaefer, D.G., and Dolan, L. (2007). An ancient mechanism controls the development of cells with a rooting function in land plants. Science *316*, 1477–1480.
49. Jang, G., and Dolan, L. (2011). Auxin promotes the transition from chloronema to caulonema in moss protonema by positively regulating PpRSL1and PpRSL2 in *Physcomitrella patens*. New Phytol. *192*, 319–327.
50. Bierfreund, N.M., Reski, R., and Decker, E.L. (2003). Use of an inducible reporter gene system for the analysis of auxin distribution in the moss *Physcomitrella patens*. Plant Cell Rep. *21*, 1143–1152.

51. Ludwig-Müller, J., Jülke, S., Bierfreund, N.M., Decker, E.L., and Reski, R. (2009). Moss (*Physcomitrella patens*) GH3 proteins act in auxin homeostasis. New Phytol. *181*, 323–338.
52. Eklund, D.M., Ståldal, V., Valsecchi, I., Cierlik, I., Eriksson, C., Hiratsu, K., Ohme-Takagi, M., Sundström, J.F., Thelander, M., Ezcurra, I., and Sundberg, E. (2010). The Arabidopsis thaliana STYLISH1 protein acts as a transcriptional activator regulating auxin biosynthesis. Plant Cell *22*, 349–363.
53. Eklund, D.M., Thelander, M., Landberg, K., Ståldal, V., Nilsson, A., Johansson, M., Valsecchi, I., Pederson, E.R.A., Kowalczyk, M., Ljung, K., et al. (2010). Homologues of the *Arabidopsis thaliana* SHI/STY/LRP1 genes control auxin biosynthesis and affect growth and development in the moss *Physcomitrella patens*. Development *137*, 1275–1284.
54. Jang, G., Yi, K., Pires, N.D., Menand, B., and Dolan, L. (2011). RSL genes are sufficient for rhizoid system development in early diverging land plants. Development *138*, 2273–2281.
55. O'Connor, D.L., Runions, A., Sluis, A., Bragg, J., Vogel, J.P., Prusinkiewicz, P., and Hake, S. (2014). A division in PIN-mediated auxin patterning during organ initiation in grasses. PLoS Comput. Biol. *10*, e1003447. http://dx.doi.org/10.1371/journal.pcbi.1003447.
56. Strepp, R., Scholz, S., Kruse, S., Speth, V., and Reski, R. (1998). Plant nuclear gene knockout reveals a role in plastid division for the homolog of the bacterial cell division protein FtsZ, an ancestral tubulin. Proc. Natl. Acad. Sci. USA *95*, 4368–4373.
57. Zazímalová, E., Murphy, A.S., Yang, H., Hoyerová, K., and Hosek, P. (2010). Auxin transporters—why so many? Cold Spring Harb. Perspect. Biol. *2*, a001552.
58. Haga, K., and Sakai, T. (2012). PIN auxin efflux carriers are necessary for pulse-induced but not continuous light-induced phototropism in *Arabidopsis*. Plant Physiol. *160*, 763–776.
59. Friml, J., Wiśniewska, J., Benková, E., Mendgen, K., and Palme, K. (2002). Lateral relocation of auxin efflux regulator PIN3 mediates tropism in *Arabidopsis*. Nature *415*, 806–809.
60. Jenkins, G.I., Courtice, G.R.M., and Cove, D.J. (1986). Gravitropic responses of wild-type and mutant strains of the moss *Physcomitrella patens*. Plant Cell Environ. *9*, 637–644.
61. Harrison, C.J., Roeder, A.H.K., Meyerowitz, E.M., and Langdale, J.A. (2009). Local cues and asymmetric cell divisions underpin body plan transitions in the moss *Physcomitrella patens*. Curr. Biol. *19*, 461–471.
62. Landberg, K., Pederson, E.R., Viaene, T., Bozorg, B., Friml, J., Jönsson, H., Thelander, M., and Sundberg, E. (2013). The MOSS *Physcomitrella patens* reproductive organ development is highly organized, affected by the two SHI/STY genes and by the level of active auxin in the SHI/STY expression domain. Plant Physiol. *162*, 1406–1419.
63. Viaene, T., Landberg, K., Thelander, M., Medvecka, E., Pederson, E., Feraru, E., Cooper, E.D., Karimi, M., Delwiche, C.F., Ljung, K., et al. (2014). Directional auxin transport mechanisms in early diverging land plants. Curr. Biol. *24*, 2786–2791.
64. Harrison, C.J., Corley, S.B., Moylan, E.C., Alexander, D.L., Scotland, R.W., and Langdale, J.A. (2005). Independent recruitment of a conserved developmental mechanism during leaf evolution. Nature *434*, 509–514.
65. Harrison, C.J., Rezvani, M., and Langdale, J.A. (2007). Growth from two transient apical initials in the meristem of *Selaginella kraussiana*. Development *134*, 881–889.
66. Harrison, C.J., and Langdale, J.A. (2010). Comment: the developmental pattern of shoot apices in *Selaginella kraussiana* (Kunze) A. Braun. Int. J. Plant Sci. *171*, 690–692.
67. Philipson, W.R. (1990). The significance of apical meristems in the phylogeny of land plants. Plant Syst. Evol. *173*, 17–38.

Current Biology, Volume *24*

Supplemental Information

Plasma Membrane-Targeted PIN Proteins

Drive Shoot Development in a Moss

Tom A. Bennett, Maureen M. Liu, Tsuyoshi Aoyama, Nicole M. Bierfreund, Marion Braun, Yoan Coudert, Ross J. Dennis, Devin O'Connor, Xiao Y. Wang, Chris D. White, Eva L. Decker, Ralf Reski, and C. Jill Harrison

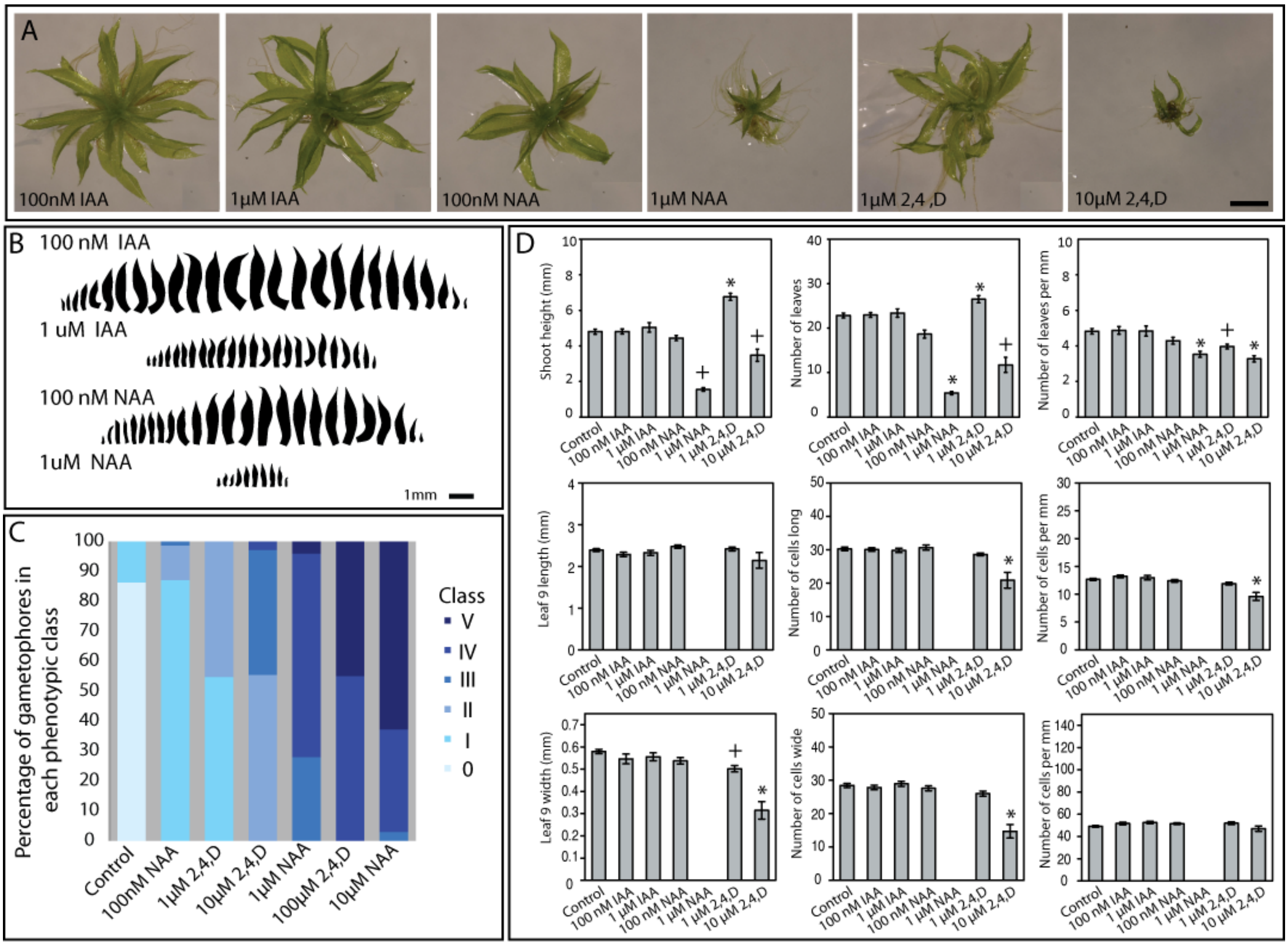

Figure S1 related to Figure 1: Different auxins have a similar developmental effects.

(a) Gametophores from plants treated with different auxins. Scale bar = 1 mm.

(b) Leaf series from plants grown on IAA and NAA. Scale bar = 1 mm.

(c) The proportion of gametophores per colony in each phenotypic class varied by treatment.

(d) Auxin accumulation at low levels promotes shoot growth and apical cell activity, but at high levels the effect is converse. Accumulation of auxin in leaves inhibits proximo-distal and medio-lateral divisions, but promotes proximo-distal expansion. Data is shown from one experimental replicate of three in which mean values and the standard error were calculated from a sample of 20 shoots. Inferences that were statistically significant in all three replicates are indicated by an asterisk, and those that were statistically significant in two replicates are indicated by a cross.

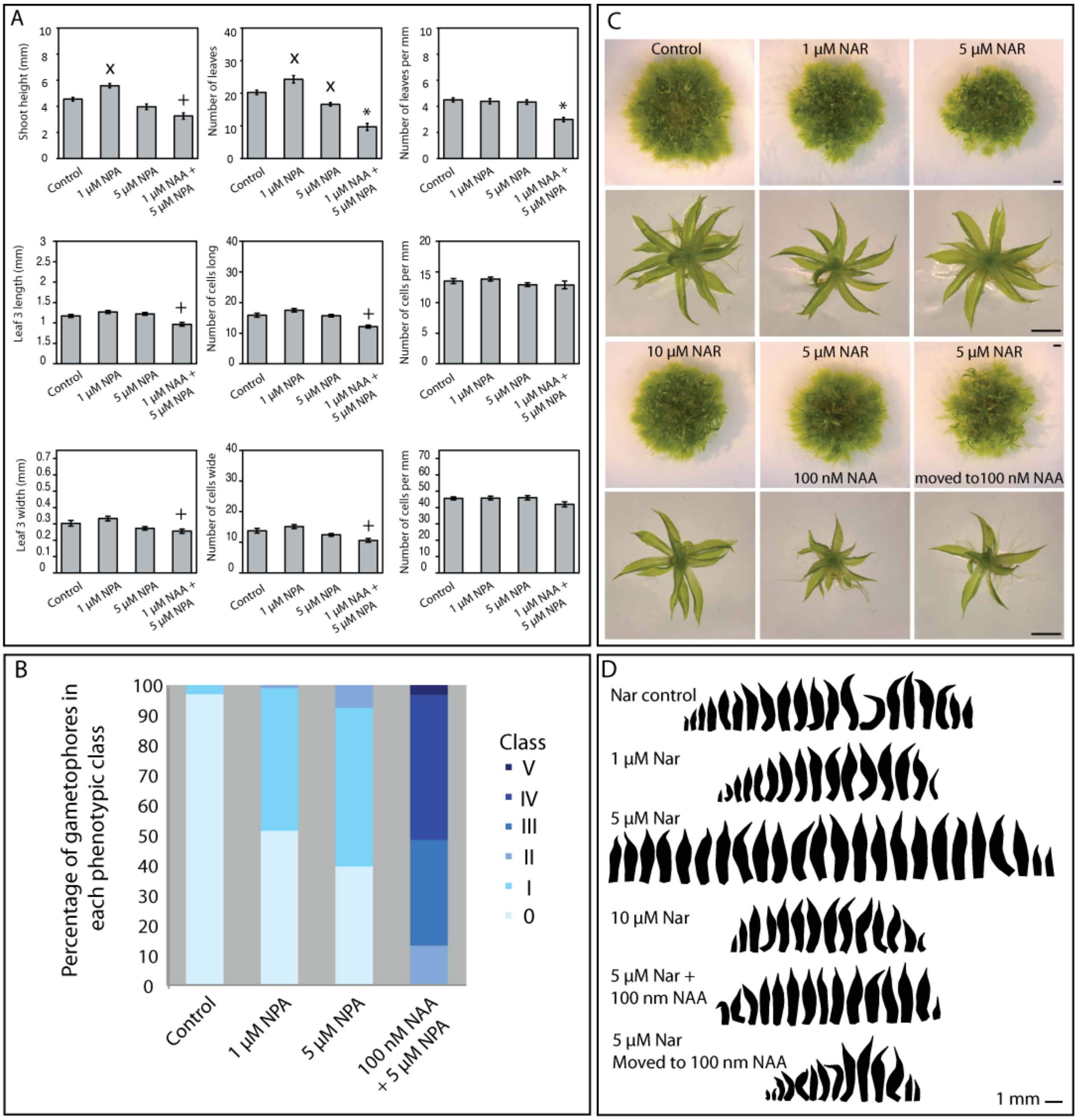

Figure S2 related to Figure 2: Effects of transport inhibition on development.

(a) Treatment with 1µM NPA phenocopies treatment with 1 µM 2,4-D and promotes shoot growth and leaf intiation, whereas 5µM NPA has a mild inhibitory effect. Addition of 100 nM NAA increases the inhibitory effect, and phenocopies 10 µM 2,4-D treatments: leaves are narrow with fewer cells by width. Data is shown from one experimental replicate of three in which mean and standard error values were calculated from a sample of 20 shoots where possible. Inferences that were statistically significant are marked by an asterisk (all three replicates), a + (two replicates), and an x (this replicate).

(b) The proportion of gametophores per colony in each phenotypic class varied by treatment.

(c) Treatment with naringenin did not strongly affect colony or gametophore development. Further addition of 100 nM NAA caused Class III defects. Scale bars = 1 mm.

(d) Whereas 5 µM naringenin (Nar) mildly promoted leaf initiation and growth, 10 µM naringenin or 5 µM naringenin with 100 nM NAA inhibited leaf initiation and growth.

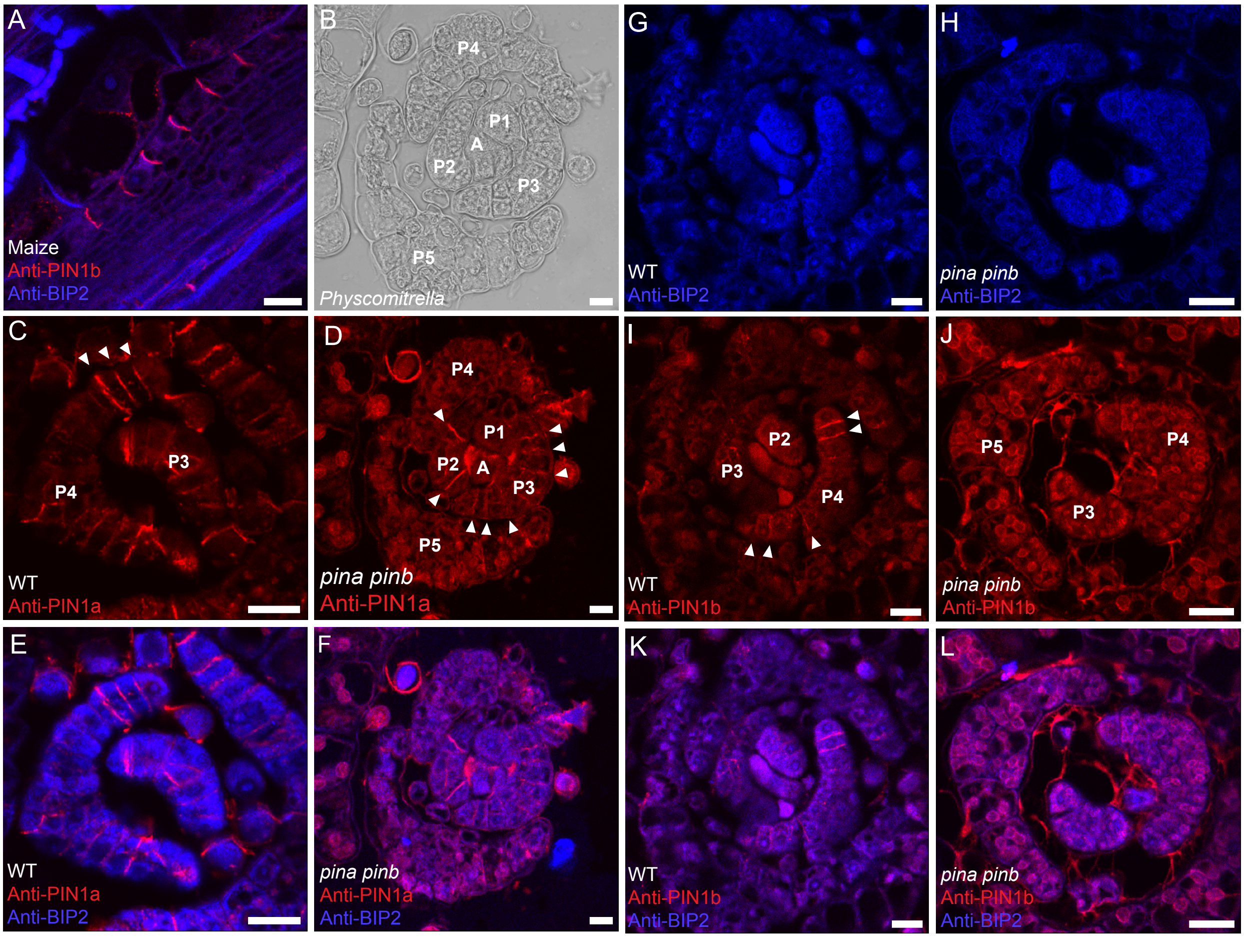

Figure S3 related to Figure 3: Immunolocalization controls.

(A) Longitudinal section showing that maize anti-PIN1b antibodies detected polar, plasma membrane targeted signal in maize leaves. Scale bar = 17.5 µm.

(B) Transverse section across a *Physcomitrella* apex showing leaves P1-P5 initiating in a spiral around the apical cell (A). Scale bar = 15 µm.

(C-F) Sections interrogated with maize anti-PIN1a antibodies in WT and *pinA pinB* mutants respectively. Note that the *pinA pinB* mutant is not null (see Figures 5 and S4), and that signal detected therefore likely reflects residual PIN accumulation. Scale bars = 15 µm.

(G-L) Immunolocalizations with the anti-PIN1b antibody showing that a similar signal distribution is detected in *Physcomitrella* with PIN1a and PIN1b antibodies. However, the signal detected is absent in *pinA pinB* mutants, suggesting that the maize anti-PIN1b antibody specifically targets PIN proteins in *Physcomitrella*. Scale bars = 15 µm.

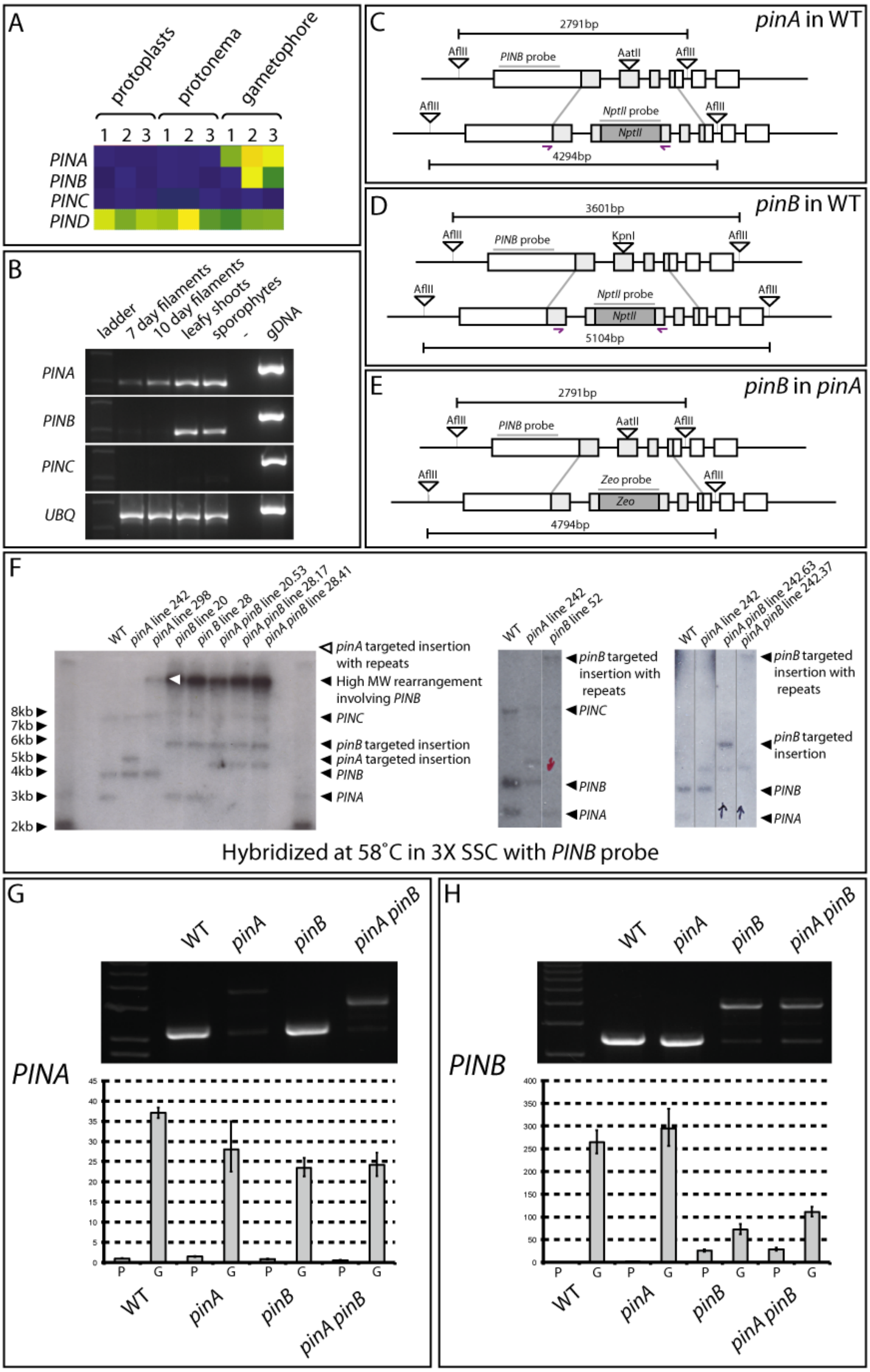

Figure S4 related to Figure 4: Expression patterns, insertion strategy and genetic analysis of *PIN* loci in WT and *pin* mutant lines.
(A) Tile display of the expression pattern of *Physcomitrella PIN* orthologues detected on a Combimatrix whole genome array.
(B) RT-PCR detected expression of *PINA* in filaments, leafy shoots and sporophytes. *PINB* was expressed strongly in leafy shoots and sporophytes. *PINC* was expressed weakly in leafy shoots and sporophytes.
(C-E) Insertion strategy for *PIN* disruption and the location of probe and primers (purple arrows) used in genetic analyses.
(F) Southern analysis with a *PINB* or resistance casette probes (not shown) confirmed targeted insertion of disruption casettes at the *PINA* and *PINB* loci in wild-type and *pinA* mutant plants.
(G, H) RT-PCR and Q-PCR showed disrupted *PINA* and *PINB* expression in *pinA*, *pinB* and *pinA pinB* mutants. Graphs show expression levels of *PINA* or *PINB* in gametophore (G) tissue relative to protonemal tissue (P) as fold change. In both *pinA* and *pinB* mutants we found two PCR amplicons; the long transcript had a c.1.7kb insertion deriving from the resistance casette at amino acid position 524 (*pinA*) or 537 (*pinB*). The short transcript from the *pinA* mutant had a 5bp deletion which introduced a stop codon at amino acid position 530, and the short transcript from the *pinB* mutant had a 9bp deletion corresponding to amino acids 543-546. All of these disruptions are in the intracellular loop region, and whilst the short *pinB* transcript could potentially generate a functional protein, it was expressed at very low levels (Fig. S4H).

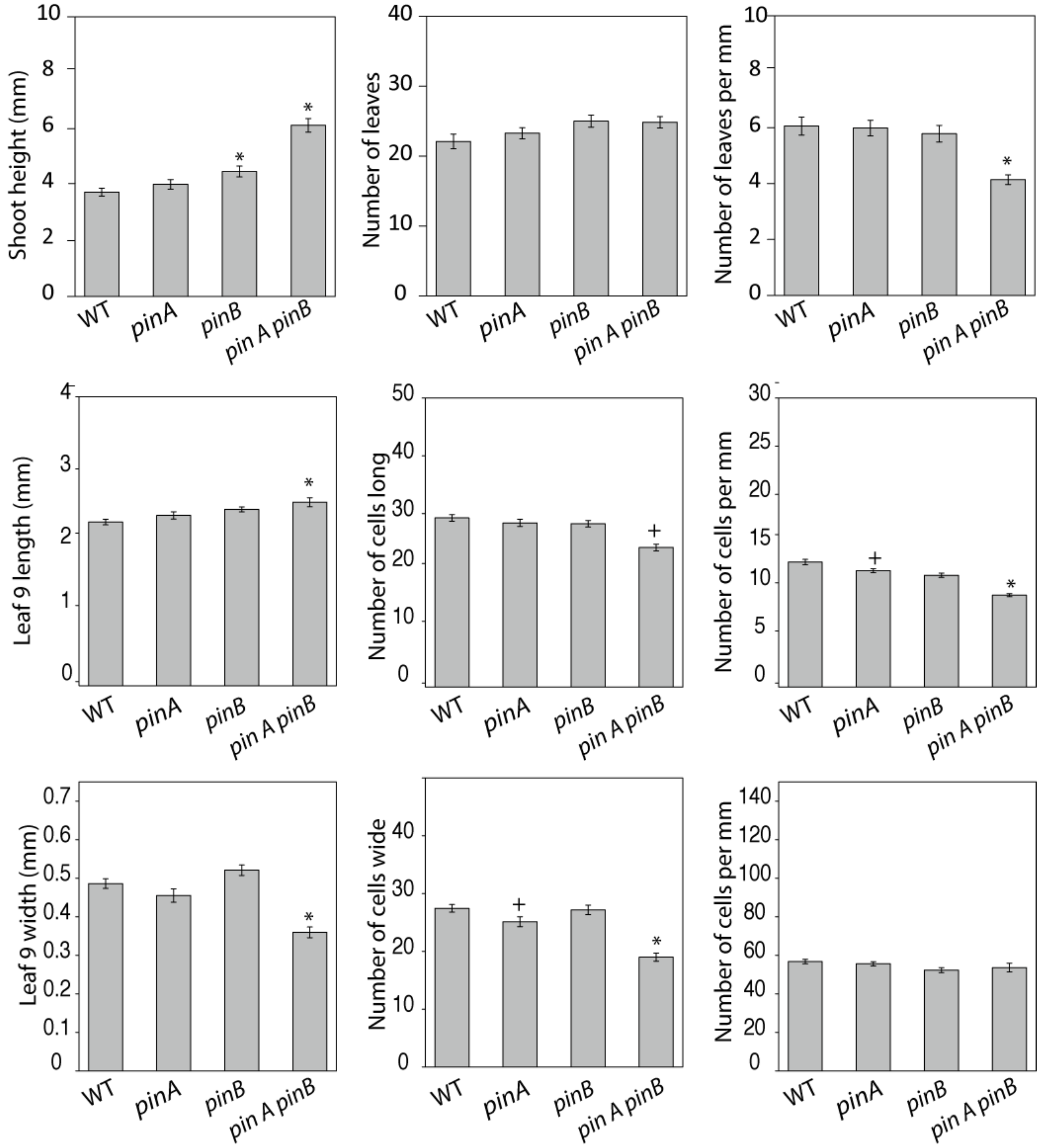

Figure S5 related to Figure 4: *pinA pinB* mutants have longer shoots with more leaves but fewer leaves per unit length than WT. *pinA pinB* mutants have longer leaves with fewer, longer cells than WT. Leaves are narrower with fewer cells by width than WT.

Data shown is from one experimental replicate of three in which mean values and the standard error were calculated from a sample of 20 shoots. Inferences that were statistically significant in all three replicates are indicates by an asterisk, and those that were statistically significant in two replicates are indicated by a cross.

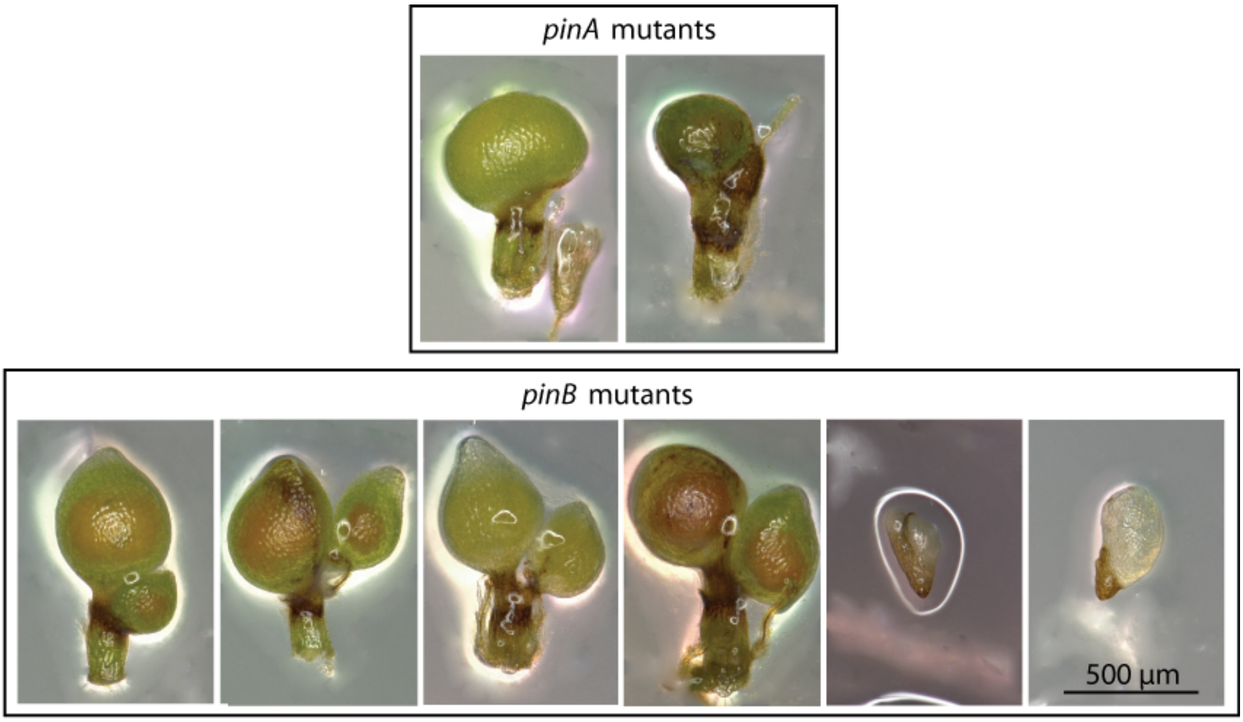

Figure S6 related to Figure 7: *pin* mutant phenotypes were variable.

Supplemental Methods.

Construct generation for insertion lines

To generate *pinA* and *pinB* disruption constructs, genomic DNA fragments corresponding to respective genes were PCR amplified using the primers Pin37 and Pin36 (*PINA*) and Pin-2C and Pin-2D (*PINB*) and cloned into pCR®4-TOPO® (Invitrogen). The *PINA* genomic fragment was disrupted by insertion of the nptII selection casette in an internal AatI restriction site (Fig. S4a). *PinB* disruptants were made by inserting the nptII casette into an internal KpnI restriction site (Fig. S4b), and double disruptants were made by replacing the nptII casette in *pinA* constructs with a zeomycin resistance casette obtained from pRT101-zeo[S1], and re-transformation into *pinB* mutant backgrounds (Fig. S4c). Prior to transformation, the insertion cassettes were released from the TOPO backbone via EcoRI digest.

Screening

Stable disruptant lines were screened for insertion as described in Fig. S4. Two *pinA* disruptants, two *pinB* disruptants, and three double disruptants had targeted insertion and loss of expression, and mutant phenotypes were shared by gene disrupted (not shown). These lines have been stored in the International Moss Stock Center as follows: GH3:GUS: IMSC#40283, *pinA* line 242: IMSC#40474, *pinA* line 298: IMSC#40475, *pinB* line 20: IMSC#40477, *pinB* line 28: IMSC#40478, *pinB* line 52: IMSC#40752, *pinA pinB* line 20.53: IMSC#40583, *pinA pinB* line 28.17: IMSC#40580, *pinA pinB* line 28.41: IMSC#40582, *pinA pinB* line 242.63: IMSC#40753, *pinA pinB* line 242.37: IMSC#40754.

Expression analyses

Evaluation of *PIN* expression by array was undertaken as described elsewhere[S2] using a cut off value of 10^4 for expression. To evaluate expression by RT-PCR, 1 µg RNA was extracted with a Qiagen RNeasy™ plant mini kit, DNAse treated then converted to cDNA with superscript III. PCR in WT (Figure S4b) was carried out using primers 5-10 listed below with an initial denaturation step of 2 minutes at 94 degrees and 30 cycles of 15 s at 94˚C, 15 s at 61˚C and 60 s at 72˚C. A final 5 minute 72˚C step was also used. PCR to screen mutants (Figure S4g, h) was carried out using primers 13-15 listed below with an initial denaturation step of 2 minutes at 98 degrees and 30 cycles of 10 s at 95˚C, 30 s at 60˚C and 2 min at 72˚C.

For Q-PCR, cDNA was synthesized using the QuantiTect Reverse Transcription Kit (QIAGEN). Quantitative RT-PCR analysis was performed using QuantiTect SYBR Green PCR Kit (QIAGEN) and CFX96 PCR machine (Bio-Rad). The following primer pairs were used: for PINA, PINA_qPCR_F1 and PINA_qPCR_R1; for PINB, PINB_qPCR_F1 and PINB_qPCR_R1, UBI, UBI-F-QPCR and UBI-R-QPCR. An initial denaturation step of 15 minutes at 94 degrees and 40 cycles of 15 s at 95˚C, 30 s at 60˚C and 30 s at 72˚C were used.

Primers used

1. Pin37: CCAGGAAGCCAAACAGCCATC
2. Pin36: GGCTGCAGCAAATACAGCTGG

3. Pin-2C: CTCCACGGGCTTCTCAAATC
4. Pin-2D: CCCAATCCCATGAACAAGCC

5. PINA-RT-F: TCCAGGAAGCCAAACAGCCAT
6. PINA-RT-R: CTCTGCCAGTTTCGGTGTCAA

7. PINB-RT-F: GTCTTGTTACTCCCGGAGGTA
8. PINB-RT-R: CTTTGCTTCGTCTTCGGGTA

9. PINC-RT-F: CGATATCTCCATTAACCTCCA
10. PINC-RT-R: GACTGAACATGGCCATCCCAA

11. Ubi-F: GCCATGCAGATCTTCGTGAA
12. Ubi-R: CTACGCAGCCAAGAACCGA

13. PINA_RT_F: TTTGGAGGTTTTCGTTTTTGG
14. PINB_RT_F: GGAGATTTGGACTGCCTCAG
15. PIN_R: TCACAGACCAAGTAATATGTAGT

16. PINA_qPCR_F1: CCCGAGAATTTGTTCCTTCA
17. PINA_qPCR_R1: CACCACTTCACAGAGCCGTA

18. PINB_qPCR_F1: AATTGTTGTGTGCGGACGTA
19. PINB_qPCR_R1: TCACCGCAGTACTGAGCATC

20. UBI-F-QPCR: CGTGCGTTGTGAGTGTTTAGA
21. UBI-R-QPCR: GCAGCCAAGAACCGATAGAT

Supplementary References

S1. Parsons, J.F. Altmann, C.K. Arrenberg, A. Koprivova, A.K. Beike, C. Stemmer, G. Gorr, R. Reski, E.L. Decker (2012): Moss-based production of asialo-erythropoietin devoid of Lewis A and other plant-typical carbohydrate determinants. Plant Biotechnology Journal *10*: 851-861

S2. Wolf, L., Rizzini, L., Stracke, R., Ulm, R. and Rensing, S. (2010) The Molecular and Physiological Responses of Physcomitrella patens to Ultraviolet-B Radiation. Plant Physiology *153*: 1123-1134
